# Abelian varieties of prescribed order over finite fields

**DOI:** 10.1007/s00208-024-03084-4

**Published:** 2025-03-06

**Authors:** Raymond van Bommel, Edgar Costa, Wanlin Li, Bjorn Poonen, Alexander Smith

**Affiliations:** 1 Department of Mathematics, Massachusetts Institute of Technology, Cambridge, MA 02139-4307 USA; 2 Present Address: School of Mathematics, Fry Building, Woodland Road, Bristol, BS8 1UG UK; 3 Centre de Recherches Mathématiques, Université de Montréal, 2920 Chemin de la tour, Montréal, QC H3T 1J4 Canada; 4 Present Address: Department of Mathematics, Washington University in St. Louis, St. Louis, MO 63130 USA; 5 Present Address: Department of Mathematics, University of California Los Angeles, Los Angeles, CA 90095 USA

**Keywords:** Primary 11G10, Secondary 11G25, 11Y99, 14G15, 14K15, 31A15

## Abstract

Given a prime power *q* and $n \gg 1$, we prove that every integer in a large subinterval of the Hasse–Weil interval $[(\sqrt{q}-1)^{2n},(\sqrt{q}+1)^{2n}]$ is $\#A({\mathbb {F}}_q)$ for some ordinary geometrically simple principally polarized abelian variety *A* of dimension *n* over ${\mathbb {F}}_q$. As a consequence, we generalize a result of Howe and Kedlaya for ${\mathbb {F}}_2$ to show that for each prime power *q*, every sufficiently large positive integer is realizable, i.e., $\#A({\mathbb {F}}_q)$ for some abelian variety *A* over ${\mathbb {F}}_q$. Our result also improves upon the best known constructions of sequences of simple abelian varieties with point counts towards the extremes of the Hasse–Weil interval. A separate argument determines, for fixed *n*, the largest subinterval of the Hasse–Weil interval consisting of realizable integers, asymptotically as $q \rightarrow \infty$; this gives an asymptotically optimal improvement of a 1998 theorem of DiPippo and Howe. Our methods are effective: We prove that if $q \le 5$, then every positive integer is realizable, and for arbitrary *q*, every positive integer $\ge q^{3 \sqrt{q} \log q}$ is realizable.

## Introduction

### Orders of abelian varieties over a finite field

By work of Weil (a consequence of [19, pp. 70–71] and [20, pp. 137–138], generalizing [4, p. 206]), if *A* is an abelian variety of dimension *n* over a finite field ${\mathbb {F}}_q$, then $\#A({\mathbb {F}}_q)$ lies in the interval

1 $$\begin{aligned} \left[ \left( q - 2q^{1/2} + 1 \right) ^n ,\; \left( q + 2q^{1/2} + 1 \right) ^n \right] . \end{aligned}$$

We prove an almost-converse (compare (1) and (3)):

#### Theorem 1.1

Fix a prime power *q*. Let $\tau (x) = x+\sqrt{x^2-1}$. Let *I* be a closed interval contained in

2 $$\begin{aligned} I_{attained }\,{:}{=}\,\bigl ( \tau (q/2 - q^{1/2} + 3/2) , \; \tau (q/2 + q^{1/2} - 1/2) \bigr ). \end{aligned}$$

For *n* sufficiently large, if *m* is a positive integer with $m^{1/n} \in I$, then there exists an *n*-dimensional abelian variety *A* with $\#A({\mathbb {F}}_q)=m$. Moreover, *A* can be chosen to be ordinary, geometrically simple, and principally polarized.

We prove Theorem 1.1 in Sect. 7.

#### Corollary 1.2

Fix a prime power *q*. Then, for *n* sufficiently large, every integer in the interval

3 $$\begin{aligned} \left[ \left( q - 2q^{1/2} + 3 - q^{-1} \right) ^n , \; \left( q + 2q^{1/2} - 1 - q^{-1}\right) ^n \right] \end{aligned}$$

is $\#A({\mathbb {F}}_q)$ for some ordinary geometrically simple principally polarized abelian variety *A* of dimension *n* over ${\mathbb {F}}_q$.

The interval (3) in Corollary 1.2 contains $[q^n,q^{n+1}]$ if *n* is large enough, so Corollary 1.2 implies the following:

#### Corollary 1.3

Fix a prime power *q*. Every sufficiently large positive integer is $\#A({\mathbb {F}}_q)$ for some ordinary geometrically simple principally polarized abelian variety *A* over ${\mathbb {F}}_q$.

Corollary 1.3 answers a question of Howe and Kedlaya, who proved that every positive integer is the order of an ordinary abelian variety over ${\mathbb {F}}_2$ [7, Theorem 1]. For effective versions, see Sect. 1.5.

#### Remark 1.4

Marseglia and Springer refined [7] to prove that every finite abelian group is isomorphic to $A({\mathbb {F}}_2)$ for some ordinary abelian variety *A* over ${\mathbb {F}}_2$ [11]. Our Corollary 1.3 combined with [11, Proposition 2.7] implies that for any fixed *q*, every cyclic group of sufficiently large order is isomorphic to $A({\mathbb {F}}_q)$ for some ordinary abelian variety *A* over ${\mathbb {F}}_q$.

Throughout, *p* denotes the characteristic of ${\mathbb {F}}_q$.

#### Remark 1.5

Theorem 1.1 can be extended to produce non-ordinary abelian varieties. First, define the *p*-rank of an *n*-dimensional abelian variety *A* over ${\mathbb {F}}_q$ to be the integer $\dim _{{\mathbb {F}}_p} A[p]({\overline{{\mathbb {F}}}}_q)$ in [0, *n*]. For example, *A* is ordinary if and only if the *p*-rank is *n*. Then Theorem 1.1 holds with “ordinary” replaced by “of prescribed *p*-rank *r*” for any $r \in [0,n]$, provided that when $r=0$, we assume $m \equiv 1 \pmod {p}$; see Remark 5.9.

#### Remark 1.6

It may be that Theorem 1.1 holds for an interval larger than $I_{attained }$. There is a largest open interval $I_{true }$ containing *q* for which Theorem 1.1 holds.

### Extreme point counts for simple abelian varieties

Other authors have studied the extreme values of $\#A({\mathbb {F}}_q)^{1/\dim A}$ without trying to realize every order in between. Following [9], let ${\mathcal {A}}_q$ be the set of simple abelian varieties over ${\mathbb {F}}_q$ up to isogeny and consider

$$\begin{aligned} I_{simple }\,{:}{=}\,\bigl [ \liminf _{A \in {\mathcal {A}}_q} \#A({\mathbb {F}}_q)^{1/\dim A} ,\; \limsup _{A \in {\mathcal {A}}_q} \#A({\mathbb {F}}_q)^{1/\dim A} \bigr ]. \end{aligned}$$

(If one did not require simplicity and take $\limsup$ and $\liminf$, then for square *q* the minimum and maximum would be achieved by elliptic curves of order $q \pm 2q^{1/2} + 1$ and their powers.) Then

$$\begin{aligned} I_{attained }\subseteq I_{true }\subseteq I_{simple }\subseteq I_{Weil }\,{:}{=}\,[ q-2q^{1/2}+1 ,\; q+2q^{1/2}+1]. \end{aligned}$$

Aubry, Haloui and Lachaud [1, Corollaries 2.2 and 2.14] and Kadets [9, Theorem 1.8] found inner and outer bounds $I_{inner },I_{outer }$ for $I_{simple }$:

4 $$\begin{aligned} \Bigl [ q - \lfloor 2q^{1/2} \rfloor + 3 ,\; q + \lfloor 2q^{1/2} \rfloor - 1 - q^{-1} \Bigr ] \subseteq I_{simple }\subseteq \Bigl [ q - \lceil 2q^{1/2} \rceil + 2 , q + \lceil 2q^{1/2} \rceil \Bigr ]. \end{aligned}$$

Our inner bound $I_{attained }$ for $I_{simple }$ improves upon $I_{inner }$, but careful consideration shows that Kadets’s argument yields a better result than he claimed, an inner bound matching our $I_{attained }$ when *q* is a square.

The following diagram shows $I_{attained }\subset I_{outer }\subset I_{Weil }$, bounded by open dots, solid dots, and vertical bars, respectively. The endpoints of $I_{true }$ and $I_{simple }$ are unknown, but they lie somewhere in the (closed) dashed intervals. 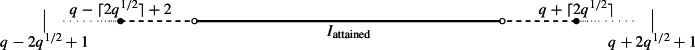


### Strategy of proof

Given an abelian variety *A* over the finite field ${\mathbb {F}}_q$, let $f_A(x) \in {\mathbb {Z}}[x]$ be the characteristic polynomial of the *q*-power Frobenius acting on a Tate module $T_\ell A$. Then $\#A({\mathbb {F}}_q)=f_A(1)$. Honda–Tate theory implies that for $f \in {\mathbb {Z}}[x]$, we have $f=f_A$ for some ordinary *n*-dimensional abelian variety *A* over ${\mathbb {F}}_q$ if and only if *f* is monic of degree 2*n* with complex roots $\alpha _1,{\bar{\alpha }}_1,\ldots ,\alpha _n,{\bar{\alpha }}_n$ satisfying $|\alpha _i|=q^{1/2}$, and *p* does not divide the coefficient of $x^n$. Therefore, as in [7], we need to find a polynomial *f* satisfying these conditions with a prescribed value of *f*(1).

One ingredient that lets us go beyond [7] is a lemma more general than [2, Lemma 3.3.1] for constructing polynomials whose roots lie on the circle $|z|=q^{1/2}$ (Lemma 3.1). Using this lemma alone, we can give a quick proof of Corollary 1.3, if we omit “geometrically simple” and “principally polarized”: see Sect. 4.

To force *A* to be geometrically simple and principally polarized, we prove that it suffices to impose certain congruence conditions on the coefficients of *f* (Proposition 5.8); unlike [2, Lemma 3.3.1], our Lemma 3.1 is robust enough to permit a wide enough range of values *f*(1) even when such congruence conditions are imposed. To prove Theorem 1.1, we start with rescaled Chebyshev polynomials similar to those in [9] (Proposition 6.2), but we improve on [9] by temporarily allowing *non-integral* real coefficients, and later making adjustments to make the coefficients integral while preserving *f*(1) and the bounds needed to apply Lemma 3.1. To obtain the widest interval of realizable values, we must adjust differently in three different ranges of exponents, and the adjustments do something more elaborate than changing one coefficient at a time; see Sect. 7.

Although we do not know if the bounds in Theorem 1.1 are sharp, Appendix A shows that the rescaled Chebyshev polynomials are asymptotically optimal for our *method*.

### Large *q* limit

So far we have discussed the possibilities for $\#A({\mathbb {F}}_q)$ for an *n*-dimensional abelian variety over a fixed finite field ${\mathbb {F}}_q$, as $n \rightarrow \infty$. We also obtain a sharp asymptotic for the possibilities for fixed *n* as $q \rightarrow \infty$:

#### Theorem 1.7

Fix $n \ge 3$. Let $\lambda _1 = 2n - \sqrt{\tfrac{2n}{n-1}}$. Then the largest interval in which every integer is $\#A({\mathbb {F}}_q)$ for some *n*-dimensional abelian variety *A* over ${\mathbb {F}}_q$ has the form

5 $$\begin{aligned} \Bigl [ q^n - \lambda _1 q^{n-1/2} + o(q^{n-1/2}) ,\; q^n + \lambda _1 q^{n-1/2} + o(q^{n-1/2}) \Bigr ] \end{aligned}$$

as $q \rightarrow \infty$ through prime powers.

#### Remark 1.8

The interval (5) is a fraction $\lambda _1/(2n)$ of the Hasse–Weil interval, approximately.

#### Remark 1.9

For $n=1$, if *q* is prime, then every integer in $[{q-2q^{1/2}{+}1}, {q{+}2q^{1/2}{+}1}]$ is $\#A({\mathbb {F}}_q)$ for some elliptic curve *A* over ${\mathbb {F}}_q$. This fails for $q=p^e$ with $e \ge 2$ because of Remark 2.2 below.

#### Remark 1.10

For $n=2$, Theorem 1.7 holds if *q* tends to $\infty$ through primes only. If instead *q* tends to $\infty$ through *non-prime* prime powers, then the constant $\lambda _1 = 2$ (asymptotically 50% of the Hasse–Weil interval) must be replaced by $\lambda _2 \,{:}{=}\,4 - 2 \sqrt{2}$ (about 29% of the Hasse–Weil interval); see Remark 8.5.

#### Remark 1.11

If we allow only *ordinary* abelian varieties, then Theorem 1.7 remains true for $n \ge 3$, as the proof will show, but for $n=2$ one must use $\lambda _2$ in place of $\lambda _1$, even if *q* is prime.

#### Remark 1.12

DiPippo and Howe proved a result implying that for any $n \ge 2$, all integers in an interval of the form (5) with $\lambda _1$ replaced by 1/2 are realized by ordinary abelian varieties [2, Theorem 1.4]. Thus Theorem 1.7 and Remark 1.11 give an asymptotically optimal improvement of their result.

Theorem 1.7 will be proved in Sect. 8.

### Effective bounds

The polynomial constructions we used to prove Theorems 1.1 and 1.7 are difficult to analyze explicitly for specific values of *q* and *n*, even when $q=3$. In Sect. 9, we give *another* construction, and this one, combined with some computations with rigorous error bounds, will allow us to prove the following theorem.

#### Theorem 1.13

Let *q* be a prime power.

- For each $q \le 5$, every positive integer is $\#A({\mathbb {F}}_q)$ for some abelian variety *A* over ${\mathbb {F}}_q$.
- For arbitrary *q*, every integer $\ge q^{3 \sqrt{q} \log q}$ is $\#A({\mathbb {F}}_q)$ for some abelian variety *A* over ${\mathbb {F}}_q$.

#### Remark 1.14

Theorem 1.13(a) is best possible: As remarked in [7], if $q \ge 7$, then 2 lies outside the union of the Hasse–Weil intervals (1).

#### Remark 1.15

Theorem 1.13(b) is best possible too, except for the constant 3, which we have not attempted to optimize. It becomes false for large *q* if 3 is replaced by any number $\delta < 1/4$, because if $n = (\delta + o(1)) \sqrt{q} \log q$, then

$$\begin{aligned} \log \frac{(\sqrt{q}-1)^{2(n+1)}}{(\sqrt{q}+1)^{2n}}&= \log q + o(1) + 2n \log \frac{\sqrt{q}-1}{\sqrt{q} + 1} \\&= \log q + o(1) + 2(\delta + o(1)) (q^{1/2} \log q) (- 2 q^{-1/2} + o(q^{-1})) \\&= (1 - 4 \delta + o(1)) \log q, \end{aligned}$$

which means that there is a large gap between the *n*th Hasse–Weil interval and the $(n+1)$st.

In Sect. 9, we will also prove the following remarks.

#### Remark 1.16

Suppose that we require *A* to be ordinary. Both statements in Theorem 1.13 remain true, except that when $q=4$ one must exclude order 3. (That 3 over ${\mathbb {F}}_4$ must be excluded follows from [9, Theorem 3.2].)

#### Remark 1.17

For $q=7$, the only positive integers not of the form $\#A({\mathbb {F}}_7)$ are 2, 14, and 17. If we require *A* to be ordinary, then 8 and 73 are the only additional integers that must be excluded.

#### Remark 1.18

Suppose that we require the characteristic polynomial of Frobenius $f_A$ to be squarefree. Then all the claims in this section remain true except that for $q=7$, the integer 16 is no longer realized.

## Honda–Tate theory

Throughout the paper, if *f* is a polynomial, $f^{[i]}$ denotes the coefficient of its degree *i* term.

### Theorem 2.1

(Honda–Tate, [5, 15, 16]) A polynomial $f \in {\mathbb {Z}}[x]$ is the characteristic polynomial of an ordinary abelian variety *A* of dimension *n* over ${\mathbb {F}}_q$ if and only if

- *f* is monic of degree 2*n*;
- all complex roots of *f* have absolute value $q^{1/2}$; and
- $$p \not \mid f^{[n]}$$.

### Remark 2.2

Let $v :{\mathbb {Q}}_p \rightarrow {\mathbb {Z}}\cup \{\infty \}$ be the *p*-adic valuation. If in Theorem 2.1 we replace (c) by both of the conditions

(c_1_): the multiplicity $\mu$ of each ${\mathbb {Q}}_p$-irreducible factor *g* in *f* is such that $\mu \, v(g(0))/v(q) \in {\mathbb {Z}}$,
(c_2_): the multiplicity of $q^{1/2}$ as a zero of *f* is even (possibly 0),

then we obtain the criterion for *f* to be the characteristic polynomial of a not-necessarily-ordinary abelian variety *A* of dimension *n* over ${\mathbb {F}}_q$. If *q* is prime, then (c_1_) holds automatically.

### Proof

As summarized in [18, Chapter 2], if *A* is a simple abelian variety over ${\mathbb {F}}_q$, then $f_A = P^e$ for some monic irreducible polynomial $P \in {\mathbb {Z}}[x]$ whose complex roots have absolute value $q^{1/2}$ and some $e \ge 1$; conversely, given such *P*, there exists a unique $e \ge 1$ such that $P^e$ is $f_A$ for a simple abelian variety *A* over ${\mathbb {F}}_q$. Moreover, the last paragraph of [18, p. 527] describes *e* as the least common denominator of certain rational numbers $i_\nu$, together with 1/2 if *P* has a real root. For $m \ge 1$, the polynomial $P^m$ satisfies (c_1_) if and only if *m* is a multiple of the denominator of *v*(*g*(0))/*v*(*q*) for each ${\mathbb {Q}}_p$-irreducible factor *g* of *P*; these ratios match Waterhouse’s second definition of $i_\nu$. For *f* of even degree, (c_2_) is equivalent to the multiplicity of $-q^{1/2}$ as a zero being even (since the roots $\ne \pm q^{1/2}$ come in complex conjugate pairs), so $P^m$ satisfies (c_2_) if and only if *P* has no real roots or *m* is even. Thus $P^m$ satisfies (c_1_) and (c_2_) if and only if *e*|*m*. This explains Remark 2.2.

Now suppose that (a), (b), (c) hold. Extend *v* to ${\overline{{\mathbb {Q}}}}_p \simeq {\mathbb {C}}$. The theory of Newton polygons implies that *f* has (at least) *n* roots $\alpha$ of valuation 0, counted with multiplicity. Their complex conjugates ${\bar{\alpha }} = q/\alpha$ are *n* roots of valuation *v*(*q*). These account for all roots. For each *g*, the value *g*(0) is a product of roots, so $v(g(0))/v(q) \in {\mathbb {Z}}$, so (c_1_) holds. Also, the multiplicity of $\pm q^{1/2}$ is 0, so (c_2_) holds. By the previous paragraph, there exists an abelian variety *A* over ${\mathbb {F}}_q$ with $f_A=f$. Finally, for an *n*-dimensional abelian variety *A* over ${\mathbb {F}}_q$, the Newton polygon definition of ordinary shows that if *A* is ordinary if and only if $p \not \mid f_A^{[n]}$. This explains Theorem 2.1. $\square$

## Roots on a circle

The following notation will be used throughout the paper:

- For $r>0$, let ${\mathbb {C}}_{\le r}$ be the closed disk $\{z \in {\mathbb {C}}: |z| \le r\}$.
- Let $$D \,{:}{=}\,{\mathbb {C}}_{\le q^{-1/2}}$$.
- For  which is a polynomial of degree $\le 2n$ (the notation implicitly depends on a choice of *n*).

To prove Theorem 1.1, we will eventually need ${\widehat{h}}$ for some polynomials *h* of degree $s>n$, in which case the two ranges of exponents of *x* overlap.

### Lemma 3.1

Let $h(z) \in {\mathbb {R}}[z]$ be a polynomial of degree $<2n$ such that *h* is nonvanishing on *D*. Then all complex roots of ${\widehat{h}}(x)$ have absolute value $q^{1/2}$.

### Proof

Since *h* is nonvanishing on *D*, the winding number of *h*(*z*) around 0 as *z* goes around the boundary $|z|=q^{-1/2}$ is 0. So the winding number of $x^n h(1/x)$ as *x* goes around the circle $|x|=q^{1/2}$ is *n*. Thus the real-valued function $2 {{\,\textrm{Re}\,}}(x^n h(1/x)) = x^n h(1/x) + q^n x^{-n} h(x/q)$ on the circle $|x|=q^{1/2}$ crosses 0 at least 2*n* times. Multiplying by $x^n$ shows that ${\widehat{h}}(x)$ has at least 2*n* roots on the circle $|x|=q^{1/2}$. It cannot have more than 2*n* roots, since $\deg {\widehat{h}} \le 2n$. $\square$

### Remark 3.2

If $h(z) = 1 + a_1 z + \cdots + a_n z^n$ with $\sum _{i=1}^n |a_i| q^{-i/2} < 1$, then $h(D) \subset \{z \in {\mathbb {C}}: {|z-1|} <1 \}$, so $0 \notin h(D)$. Thus Lemma 3.1 subsumes [2, Lemma 3.3.1], which appears also (with a different proof) as [7, Lemma 2]. The feature of Lemma 3.1 that allows us to obtain stronger results is that $\{h: 0 \notin h(D)\}$ is closed under multiplication, a natural property given that one can take products of abelian varieties.

### Remark 3.3

The polynomials ${\widehat{h}}(x)$ produced by Lemma 3.1 are squarefree.

### Remark 3.4

Applying Lemma 3.1 to *h*(*rx*) as $r \rightarrow 1^-$ shows that the hypothesis could be weakened to assume only that *h* is nonvanishing on the *interior* of *D*.

For use in the proof of Lemma 7.1, we record the following result.

### Lemma 3.5

Let $R \in {\mathbb {C}}[z]$ be a polynomial with no zeros inside *D*. Then

6 $$\begin{aligned} |R(1)| \le q^{(\deg R)/2} |R(1/q)|. \end{aligned}$$

### Proof

By multiplicativity in *R*, we may assume that $R(z) = z-w$ for some $w \in {\mathbb {C}}$ with $|w| \ge q^{-1/2}$. We must prove $|(1-w)/(1/q-w)| \le q^{1/2}$. The Möbius transformation $M(z) \,{:}{=}\,(1-z)/(1/q-z)$ maps the circle $|z|=q^{-1/2}$ to a complex-conjugation-invariant circle passing through $M(\pm q^{-1/2}) = \pm q^{1/2}$, and it maps the exterior to the interior since $M(\infty )=1$. $\square$

## Abelian varieties of all sufficiently large orders

As promised in the introduction, we now give a quick proof of Corollary 1.3 with “geometrically simple” and “principally polarized” removed.

### Theorem 4.1

Fix a prime power *q* and a closed interval $I \subset {\mathbb {R}}_{>0}$. For $n \gg 1$, each integer $m \in q^n I$ is $\#A({\mathbb {F}}_q)$ for some ordinary abelian variety *A* of dimension *n* over ${\mathbb {F}}_q$ whose characteristic polynomial is squarefree.

### Proof

For $k \ge 1$, let ${\mathcal {J}}_k$ be the set of power series of the form $1+a_k z^k + a_{k+1} z^{k+1} + \cdots$ with integer coefficients in $[-q/2,q/2]$. Choose *k* such that $1 - \sum _{r \ge k} \lfloor q/2 \rfloor q^{-r/2} \ge 1/2$; then $|j(w)| \ge 1/2$ for all $j \in {\mathcal {J}}_k$ and $w \in D$. Writing real numbers in base *q* using digits in $[-q/2,q/2]$ shows that $\{j(1/q): j \in {\mathcal {J}}_k\}$ contains a neighborhood of 1, say $[1-\epsilon ,1+\epsilon ]$ for some $\epsilon >0$. Choose *N* such that $[(1-\epsilon )^N,(1+\epsilon )^N] \supset I$. Then, given $m \in q^n I$, we may choose $j \in {\mathcal {J}}_k$ with $j(1/q)^N = m/q^n$. Write $j^N = h_0 + h_1$ such that $h_0 \in 1 + z^k {\mathbb {Z}}[z]$ is of degree $\le n$, and $h_1 \in z^{n+1}{\mathbb {Z}}[[z]]$. Let $E = m - {\widehat{h}}_0(1)$. Let

7 $$\begin{aligned} h = h_0 + (E/2) z^n + s (z^{n-1}-((q+1)/2) z^n), \end{aligned}$$

where $s \in \{0,1\}$ is chosen so that *p* does not divide the coefficient of $x^n$ in

$$\begin{aligned} {\widehat{h}} = {\widehat{h}}_0 + E x^n + s (x^{n+1} - (q+1) x^n + q x^{n-1}). \end{aligned}$$

Then ${\widehat{h}}$ is a monic polynomial of degree 2*n* in ${\mathbb {Z}}[x]$ and ${\widehat{h}}(1) = {\widehat{h}}_0(1) + E = m$. The conclusion follows from Lemma 3.1, Theorem 2.1, and Remark 3.3 if we can show that *h* is nonvanishing on *D*. We will do so by estimating the error in the approximations $h \approx h_0 \approx j^N$.

Since *j* has bounded coefficients, induction on *N* shows that $|(j^N)^{[r]}| = O(r^{N-1})$ as $r \rightarrow \infty$, uniformly for $j \in {\mathcal {J}}_k$. Thus

$$\begin{aligned} |h_0(1)|&= \biggl |\sum _{r=0}^n (j^N)^{[r]} \biggr |\le \sum _{r=0}^{n} O(r^{N-1}) = O(n^N), \\ |h_1(1/q)|&= \biggl |\sum _{r=n+1}^\infty (j^N)^{[r]} q^{-r} \biggr |\le \sum _{r=n+1}^{\infty } O(r^{N-1}) q^{-r} = O(n^{N-1} q^{-n-1}), \\ |h_1(w)|&= O(n^{N-1} q^{-(n+1)/2}) for all \,\, w \in D,\text { similarly, and} \\ |E|&= |m - {\widehat{h}}_0(1)| = |q^n j(1/q)^N - (q^n h_0(1/q)+ h_0(1))|\\&\le |q^n h_1(1/q)| + |h_0(1)| = O(n^N). \end{aligned}$$

Substituting $h_0 = j^N-h_1$ into (7) yields

$$\begin{aligned} h(z) = j(z)^N - h_1(z) + (E/2) z^n + s (z^{n-1}-((q+1)/2) z^n), \end{aligned}$$

For $w \in D$, we proved $|j(w)| \ge 1/2$ and $|h_1(w)| = O(n^{N-1} q^{-(n+1)/2})$, so

$$\begin{aligned} |h(w)| \ge 2^{-N} - O(n^{N-1} q^{-(n+1)/2}) - O(n^N q^{-n/2}) - O(q \cdot q^{-n/2}) > 0 \end{aligned}$$

if *n* is large enough. $\square$

By [15, Theorem 2c], an abelian variety *A* over ${\mathbb {F}}_q$ has a squarefree characteristic polynomial if and only if its endomorphism ring $\text {End}_{{\mathbb {F}}_q} A$ is commutative. So Theorem 4.1 implies the following.

### Corollary 4.2

Fix a prime power *q*. Every sufficiently large positive integer is $\#A({\mathbb {F}}_q)$ for some ordinary abelian variety *A* over ${\mathbb {F}}_q$ with commutative endomorphism ring.

### Proof

Apply Theorem 4.1 with $I=[1,q]$. $\square$

## A congruence condition forcing geometric simplicity and the existence of principal polarizations

The goal of this section is Proposition 5.8, which provides a congruence condition on the characteristic polynomial of an abelian variety *A* over ${\mathbb {F}}_q$ which guarantees that *A* is geometrically simple and isogenous to a principally polarized abelian variety. Moreover, the congruence condition will be compatible with prescribing $\#A({\mathbb {F}}_q)$. The lemmas in this section are used only to prove Proposition 5.8.

### Lemma 5.1

For every prime power *q*, prime $\ell \ge 7$ not dividing *q*, and integer $n \ge 1$, there exists $j(x) \in {\mathbb {F}}_\ell [x]$ such that *j*(*x*) and $x^n j(q/x)$ are relatively prime irreducible polynomials of degree *n* not vanishing at 1.

### Proof

If $n=1$, choose $j(x)=x-a$ where $a \in {\mathbb {F}}_\ell - \{0,1,q,\pm \sqrt{q}\}$. If $n=2$, let *j*(*x*) be the minimal polynomial of an element $\alpha \in {\mathbb {F}}_{\ell ^2}^\times - {\mathbb {F}}_\ell ^\times$ such that $\alpha \ne q/\alpha$ and $\alpha ^\ell \ne q/\alpha$; there are at least $(\ell ^2-\ell ) - 2 - (\ell +1) > 0$ such elements $\alpha$.

Now suppose that $n \ge 3$. Let $\alpha$ be a generator of the multiplicative group ${\mathbb {F}}_{\ell ^n}^\times$. Let *j*(*x*) be the minimal polynomial of $\alpha$ over ${\mathbb {F}}_\ell$. If *j*(*x*) and $x^n j(q/x)$ are not relatively prime, then $\alpha ^{\ell ^a} = q/\alpha$ for some $a \in \{0,1,\ldots ,n-1\}$. Then $\alpha ^{(\ell -1)(\ell ^a + 1)} = q^{\ell -1} = 1$ in ${\mathbb {F}}_{\ell ^n}$, so $\ell ^n-1$ divides $(\ell -1)(\ell ^a + 1)$, contradicting $0< (\ell -1)(\ell ^a + 1) < \ell ^n-1$. $\square$

Call a degree 2*n* polynomial *f* over a ring *q*-symmetric if $f^{[i]} = q^{n-i} f^{[2n-i]}$ for $i=0,\ldots ,n-1$. Over a ring in which *q* is not a zerodivisor, *f* is *q*-symmetric if and only if $q^n f(x) = x^{2n} f(q/x)$. By work of Weil, the characteristic polynomial of an abelian variety over ${\mathbb {F}}_q$ is *q*-symmetric.

### Lemma 5.2

Let *q* be a prime power, let $\ell \ge 7$ be a prime not dividing *q*, let $n \in {\mathbb {Z}}_{\ge 1}$, and let $m \in {\mathbb {Z}}$. Suppose that $d_1,\ldots ,d_r$ are positive integers summing to *n* such that 1 appears exactly once or twice among $d_1,\ldots ,d_r$ and every other positive integer appears at most once. Then there exists a monic *q*-symmetric polynomial $g(x) \in {\mathbb {F}}_\ell [x]$ such that

- $$g(1) = m \bmod \ell $$,
- the roots of *g* form *n* *distinct* multiset pairs $\{\alpha ,q/\alpha \}$; and
- the Frobenius element of ${{\,\textrm{Gal}\,}}({\overline{{\mathbb {F}}}}_\ell /{\mathbb {F}}_\ell )$ acts on these *n* pairs as a permutation consisting of cycles of lengths $d_1,\ldots ,d_r$.

### Proof

For each *i* with $d_i \ge 2$, let $j_i(x)$ be the polynomial of degree $d_i$ provided by Lemma 5.1, and let $g_i(x) = j_i(x) \cdot x^{d_i} j_i(q/x)$. For each *i* with $d_i=1$, let $g_i(x) = x^2-a_i x+q$ for some $a_i \in {\mathbb {F}}_\ell$ to be determined. Each $g_i$ is *q*-symmetric. Since each $j_i$ is irreducible, the *q*-symmetric polynomial $g(x) \,{:}{=}\,\prod _{i=1}^r g_i(x)$ gives the correct cycle type, and its irreducible factors are distinct, except possibly for the factors of the $g_i$ for which $d_i=1$.

If exactly one $d_i$ equals 1, then there is a unique choice of $a_i$ in ${\mathbb {F}}_\ell$ that makes $g(1) = m \bmod \ell$. If $d_i$ and $d_j$ both equal 1 (with $i \ne j$), then there are at least $\ell -1$ choices for $(a_i,a_j)$ that make $g(1) = m \bmod \ell$ and at most two of these satisfy $a_i=a_j$; thus we can ensure $g(1) = m \bmod \ell$ while making *g* separable. $\square$

### Lemma 5.3

For every prime power *q*, integer *m*, prime $\ell > q+2\sqrt{q}+1$, and integer $n \ge 8\sqrt{q}+5$, there exists a monic *q*-symmetric polynomial $g(x) \in {\mathbb {F}}_\ell [x]$ of degree 2*n* such that $g(1)=m \bmod \ell$ and *g*(*x*) has no factor of the form $x^2-{\overline{a}}x+q$ with $a \in {\mathbb {Z}}$ and $|a| \le 2\sqrt{q}$.

### Proof

Since $\ell > q+2\sqrt{q}+1$, none of the polynomials $x^2-{\overline{a}}x+q$ vanish at $1\bmod \ell$. Lagrange interpolation provides a monic degree *n* polynomial $j(x) \in {\mathbb {F}}_\ell [x]$ such that $j(0)=1$, $j(1)=m$, $j(q)=1$, and $j(\alpha )=1$ for every root $\alpha \in {\overline{{\mathbb {F}}}}_\ell$ of the quadratic polynomials $x^2-{\overline{a}}x+q$ (the number of values to specify is at most $3+2(4\sqrt{q}+1) \le n$). Take $g(x) \,{:}{=}\,j(x) \cdot x^n j(q/x)$. $\square$

### Lemma 5.4

Let $n \ge 3$. A subgroup *G* of $S_n$ containing an $(n-1)$-cycle, an $(n-2)$-cycle, and a 2-cycle is either $S_n$ or the stabilizer $S_{n-1}$ of the fixed point of the $(n-1)$-cycle.

### Proof

Without loss of generality, the fixed point of the $(n-1)$-cycle is *n*. If $G \le S_{n-1}$, then *G* acts on $\{1,2,\ldots ,n-1\}$ transitively (because of the $(n-1)$-cycle) and primitively (because of the $(n-2)$-cycle); a primitive subgroup of $S_{n-1}$ containing a 2-cycle is the whole group $S_{n-1}$ [8, Theorem 8.17]. Otherwise *G* acts on $\{1,\ldots ,n\}$ transitively (because the orbit of 1 is larger than $\{1,2,\ldots ,n-1\}$) and primitively (because of the $(n-1)$-cycle), and then the 2-cycle forces $G = S_n$. $\square$

### Lemma 5.5

Let $n \ge 5$. Let *A* be an *n*-dimensional abelian variety over ${\mathbb {F}}_q$. Write $f_A(x) = x^n R(x+q/x)$ for some monic degree *n* polynomial $R(x) \in {\mathbb {Z}}[x]$. If the Galois group of *R* is $S_n$ or the stabilizer $S_{n-1}$ of a point, then *A* is either geometrically simple or a product of geometrically simple abelian varieties over ${\mathbb {F}}_q$ of dimensions $n-1$ and 1.

### Proof

If *A* is isogenous to $A_1 \times A_2$ over ${\mathbb {F}}_q$, then *R* factors correspondingly into $R_1 R_2$. Since *R* is either irreducible or a product of irreducible polynomials of degrees 1 and $n-1$, the abelian variety *A* is either simple or a product of simple abelian varieties of dimensions 1 and $n-1$. Let $A'$ be the simple factor of dimension $d \in \{n,n-1\}$, and define $R'$ accordingly.

Suppose that $A'$ is not geometrically simple. Let $r>1$ be such that $A'_{{\mathbb {F}}_{q^r}}$ is not simple. Then $f_{A'}$ has roots $\alpha ,\beta \in {\overline{{\mathbb {Q}}}}$ giving rise to distinct roots $\alpha +q/\alpha \ne \beta +q/\beta$ of $R'$ such that $\alpha ^r = \beta ^r$. Now $\beta = \zeta \alpha$ for some root of unity $\zeta$. Thus the extension ${\mathbb {Q}}(\alpha , \zeta ) \supset {\mathbb {Q}}(\alpha +q/\alpha )$, being the compositum of two abelian extensions, is abelian, so its subfield ${\mathbb {Q}}(\alpha +q/\alpha , \beta +q/\beta )$ is Galois over ${\mathbb {Q}}(\alpha +q/\alpha )$, contradicting the fact that $S_{d-2}$ is not normal in $S_{d-1}$. $\square$

### Lemma 5.6

For every prime power $q=p^e$, prime $\lambda \ge 7$ such that *q* is a nonzero square modulo $\lambda$, and integers $n \ge 5$ and *m*, there exists a monic *q*-symmetric degree 2*n* polynomial $g(x) \in ({\mathbb {Z}}/\lambda ^2 {\mathbb {Z}})[x]$ with $g(1) = m \bmod {\lambda ^2}$ such that if *A* is a simple abelian variety over ${\mathbb {F}}_q$ with $f_A \bmod \lambda ^2$ equal to *g*, then the isogeny class of *A* contains a principally polarized abelian variety.

### Proof

By Hensel’s lemma, we can choose $s \in {\mathbb {Z}}$ such that the discriminant of $x^2-sx+q$ is $0 \bmod \lambda$ but nonzero mod $\lambda ^2$. Replace *s* by $-s$, if necessary, to make $q+1-s \not \equiv 0 \pmod {\lambda }$. Choose a monic irreducible polynomial $S(x) \in {\mathbb {F}}_{\lambda }[x]$ of degree $n-3$. Choose $a,b \in {\mathbb {F}}_\lambda$ such that the polynomial ${\bar{R}} \,{:}{=}\,(x-s)(x-a)(x-b)S(x) \in {\mathbb {F}}_\lambda [x]$ is separable and ${\bar{R}}(q+1) = m \bmod \lambda$; this amounts to choosing two elements of ${\mathbb {F}}_\lambda$ (namely, $q+1-a$ and $q+1-b$) with prescribed product, not equal to $q+1-s$ or each other, which is possible because $\lambda -1>4$. Let $R \in ({\mathbb {Z}}/\lambda ^2{\mathbb {Z}})[x]$ be a lift of ${\bar{R}}$ such that $R(s)=0$ and $R(q+1)=m$ in ${\mathbb {Z}}/\lambda ^2{\mathbb {Z}}$. Let $g(x) = x^n \, R(x+q/x) \in ({\mathbb {Z}}/\lambda ^2{\mathbb {Z}})[x]$.

Suppose that *A* is a simple abelian variety over ${\mathbb {F}}_q$ such that $f_A \bmod \lambda ^2$ is *g*. Since *A* is simple, $f_A$ is a power of an irreducible polynomial [18, Chapter 2], but its reduction $g \bmod \lambda$ has some simple roots (for example, the roots of $x^{n-3} S(x+q/x)$), so $f_A$ must be irreducible, of degree 2*n*. Let $\pi \in {\overline{{\mathbb {Q}}}}$ be a root of $f_A$. Let $K = {\mathbb {Q}}(\pi )$ and $K^+ = {\mathbb {Q}}(\pi +q/\pi )$, so *K* is a CM field and $K^+$ is its maximal totally real subfield. Since the minimal polynomial of $\pi +q/\pi$ reduces to ${\bar{R}}$, the extension $K^+/{\mathbb {Q}}$ is unramified above $\lambda$. On the other hand, $K/K^+$ is ramified at the prime above $\lambda$ corresponding to the root *s* of *g*, because the discriminant of $x^2-sx+q$ has odd valuation 1. By [6, Theorem 1.1], the isogeny class of *A* contains a principally polarized abelian variety. $\square$

### Lemma 5.7

For any prime power $q = p^e$, there exists a prime $\lambda$ such that $7\le \lambda <q^3 and\, q \,is \,a \,nonzero \,square \,mod\, \lambda .$

### Proof

We will choose $\lambda$ to be a prime factor of $u^2-q$ for some integer *u* in $[\sqrt{q}-30,\sqrt{q}+30]$ chosen so that $u^2-q \ne \pm 1$ and $u^2-q$ is not divisible by 2, 3, or 5. There are at least six integers *u* in $[\sqrt{q}-30,\sqrt{q}+30]$ such that $u^2-q$ is not divisible by 2, 3, or 5. At most two of them satisfy $u^2-q=\pm 1$; among the other four are two differing by 30, and one of them is prime to *p*. Thus *u* can be found. Then $\lambda \ne 2,3,5,p$, and $\lambda \le (\sqrt{q}+30)^2-q$, which is less than $q^3$, except for $q<11$ for which we instead compute an explicit $\lambda$. $\square$

### Proposition 5.8

Given a prime power *q*, there exists a positive integer *L* such that for any integers $n \gg 1$ and *m*, there exists a monic *q*-symmetric polynomial $g(x) \in ({\mathbb {Z}}/L{\mathbb {Z}})[x]$ of degree 2*n* with $g(1) = m \bmod L$ such that any *n*-dimensional abelian variety *A* over ${\mathbb {F}}_q$ whose characteristic polynomial reduces modulo *L* to *g*(*x*) is ordinary, geometrically simple, and isogenous to a principally polarized abelian variety. Moreover, we may choose $L < q^{23}$.

### Proof

Let $\lambda$ be as in Lemma 5.7. Let $L = p \lambda ^2 \ell _0 \ell _1 \ell _2 \ell _3$, where *p* is the characteristic, and $p,\lambda ,\ell _0,\ldots ,\ell _3$ are distinct primes such that $\ell _0 > q+2\sqrt{q}+1$ and $\ell _i \ge 7$ for $i=1,\ldots ,3$. Suppose that $n \ge 8\sqrt{q}+5$. Let $\gamma (x) \in {\mathbb {F}}_p[x]$ be a monic *q*-symmetric polynomial of degree 2*n* such that $\gamma (1) = m \bmod p$; add $x^{n+1}-x^n$, if necessary, to make $\gamma ^{[n]} \ne 0 \bmod {p}$ (here *q*-symmetry means only that $\gamma ^{[i]}=0$ for $i<n$). Let $g_\lambda (x) \in ({\mathbb {Z}}/\lambda ^2{\mathbb {Z}})[x]$ be as in Lemma 5.6. Apply Lemma 5.3 to produce a polynomial $g_0(x) \in {\mathbb {F}}_{\ell _0}[x]$. Apply Lemma 5.2 to produce polynomials $g_i(x) \in {\mathbb {F}}_{\ell _i}[x]$ for $i=1,2,3$ corresponding to the partitions

- $$(n-1,1)$$
- $$(n-2,1,1)$$
- $(n-3,2,1)$ if *n* is even; and $(n-4,2,1,1)$ *n* is odd,

respectively. Let $g \in ({\mathbb {Z}}/L{\mathbb {Z}})[x]$ be the monic *q*-symmetric polynomial of degree 2*n* reducing to $\gamma$, the $g_i$, and $g_\lambda$.

Suppose that *A* is an *n*-dimensional abelian variety over ${\mathbb {F}}_q$ such that $f_A(x) \bmod L = g(x)$. Write $f_A(x) = x^n R(x+q/x)$. Let $G \le S_n$ be the Galois group of *R*, which encodes the action of ${{\,\textrm{Gal}\,}}({\overline{{\mathbb {Q}}}}/{\mathbb {Q}})$ on the pairs $\{\alpha ,q/\alpha \}$ of roots of *F*. By choice of $g_1,g_2,g_3$, the group *G* contains permutations $\sigma _1,\sigma _2,\sigma _3$ whose cycle types are given by the partitions above. Raising $\sigma _3$ to the power $n-3$ or $n-4$, whichever is odd, produces a 2-cycle. By Lemma 5.4, *G* is $S_n$ or $S_{n-1}$. By Lemma 5.5, *A* is either geometrically simple or a product of geometrically simple abelian varieties over ${\mathbb {F}}_q$ of dimensions $n-1$ and 1. In the second case, $f_A(x)$ would have a factor $x^2-ax+q$ for some integer *a* with $|a| \le 2\sqrt{q}$, which is ruled out by the choice of $g_0$. Thus *A* is geometrically simple. Since $\gamma ^{[n]} \ne 0 \bmod p$, *A* is ordinary. By Lemma 5.6, *A* is isogenous to a principally polarized abelian variety.

In proving $L < q^{23}$, the worst case is $q=2$, in which case we take $L=2 \cdot 7^2 \cdot 11 \cdot 13 \cdot 17 \cdot 19 < 2^{23}$. $\square$

### Remark 5.9

It is not hard to adapt Proposition 5.8 for the purpose of constructing abelian varieties of prescribed order that have *prescribed p-rank*. Namely, one can prove that it suffices to impose congruences modulo $pq^2$ on the coefficients of a *q*-symmetric monic degree 2*n* polynomial *f* to guarantee that its Newton polygon is the lowest Newton polygon corresponding to *p*-rank *r* and that its segments of slope in $[-1/2,0]$ correspond to ${\mathbb {Q}}_p$-irreducible factors, in which case the other segments do too by *q*-symmetry, so that (c_1_) in Remark 2.2 is satisfied; moreover one can make these congruences compatible with $f(1) \equiv m \pmod {pq^2}$, provided that, in the case $r=0$, one has $m \equiv 1 \pmod {p}$. This last hypothesis is necessary: if *A* has *p*-rank 0, then all roots of $f_A$ have positive *p*-adic valuation, so $\#A({\mathbb {F}}_q) \equiv 1 \pmod {p}$.

## Chebyshev polynomials

Choose the branch of $\sqrt{z^2-1}$ on ${\mathbb {C}}-[-1,1]$ that is $z + o(1)$ as $z \rightarrow \infty$. Let $\tau (z) = z + \sqrt{z^2-1}$. Define the *d*th Chebyshev polynomial

8 $$\begin{aligned} T_d(z) = \frac{1}{2}\left( \left( z + \sqrt{z^2 - 1}\right) ^d + \left( z - \sqrt{z^2 - 1}\right) ^d\right) = (\tau (z)^d + \tau (z)^{-d}) / 2. \end{aligned}$$

### Lemma 6.1

For a suitable choice of *d*th root, the functions $T_d(z)^{1/d}/z$ and $\tau (z)/z$ extend to holomorphic functions on ${\mathbb {P}}^1({\mathbb {C}}) {\setminus } [-1,1]$, and $T_d(z)^{1/d}/z \rightarrow \tau (z)/z$ uniformly on any compact subset of that domain as $d \rightarrow \infty$.

### Proof

Since $$\tau $$ is nonvanishing with a simple pole at $$\infty $$, the maximum modulus principle applied to $$1/\tau $$ shows that $$|\tau (z)|$$ is minimized as *z* approaches $$[-1,1]$$, in which case $$|\tau (z)| \rightarrow 1$$. Thus $$|\tau (z)| > 1$$ on $${\mathbb {P}}^1({\mathbb {C}}) {\setminus } [-1,1]$$, so $$T_d(z) \ne 0$$ on $${\mathbb {C}}- [-1,1]$$. Also, as $$z \rightarrow \infty $$, we have $$T_d(z) = z^d + (lower order terms )$$, so we can choose a *d*th root with $$T_d(z)^{1/d} = z + (lower order terms )$$. The uniform convergence claim now follows from $$T_d(z)/z^d = \tfrac{1}{2}z^{-d}(\tau (z)^d + \tau (z)^{-d})$$. $$\square $$

Recall from Sect. 3 that *D* is the closed disk ${\mathbb {C}}_{\le q^{-1/2}}$ of radius $q^{-1/2}$.

### Proposition 6.2

Let *I* be a closed interval contained in $I_{attained }$ (see (2)). Then for even $d \gg 1$, there exists a degree *d* polynomial $P(z) \in {\mathbb {R}}[z]$ such that

- $$P(0)=1$$;
- *P* is positive on ${\mathbb {R}}$;
- $|P(w)|^{1/d} \ge q^{-1/4}$ for all $w \in D$; and
- $$(q P(1/q)^{2/d}, q P(-1/q)^{2/d})$$ contains *I*.

### Remark 6.3

In Appendix A, we use potential theory to prove that Proposition 6.2 is optimal in the sense that it fails if $I_{attained }$ is enlarged.

### Proof

For $\epsilon >0$ to be specified later, let

$$\begin{aligned} \ell (z)&= (q^{1/2}/2) z - (q^{1/2}-1), \\ f_d(z)&= 2 q^{-d/4} z^{d/2} \, T_{d/2}(\ell (z+1/z)), \\ P(z)&= f_d((1-\epsilon )q^{1/2} z). \end{aligned}$$

- The leading coefficient of $T_{d/2}$ is $2^{d/2-1}$, so $f_d(0) = 2 q^{-d/4} 2^{d/2-1} (q^{1/2}/2)^{d/2} = 1$ and $P(0)=f_d(0)=1$.
- The roots of $$T_{d/2}$$ are in $$[-1,1)$$, and $$\ell ^{-1}([-1,1)) \subset (-2,2)$$, so all the roots of $$f_d(z)$$ are on the unit circle and not at $$\pm 1$$. Thus $$f_d$$ does not change sign on $${\mathbb {R}}$$. Since $$f_d(0)>0$$, the sign is positive. Thus *P* is positive on $${\mathbb {R}}$$.
- The function $(1-\epsilon )q^{1/2}z$ maps *D* to ${\mathbb {C}}_{\le 1-\epsilon }$, so we need to prove that $|f_d|^{1/d} \ge q^{-1/4}$ on ${\mathbb {C}}_{\le 1-\epsilon }$. First, $z T_{d/2}(\ell (z+1/z))^{2/d}$ is the product of the polynomial $z \, \ell (z+1/z)$ and holomorphic function $T_{d/2}(\ell (z+1/z))^{2/d}/\ell (z+1/z)$ on ${\mathbb {C}}_{\le 1-\epsilon }$, so Lemma 6.1 implies that
  9 $$\begin{aligned} |f_d(z)|^{1/d} \rightarrow q^{-1/4} |z|^{1/2} \left| \tau (\ell (z+1/z)) \right| ^{1/2} \end{aligned}$$
  uniformly for $z \in {\mathbb {C}}_{\le 1-\epsilon }$. The function $z \, \tau (\ell (z+1/z))$ is holomorphic, nonconstant, and nonvanishing on ${\mathbb {C}}_{<1}$, and it extends to a continuous function on ${\mathbb {C}}_{\le 1}$ having absolute value $\ge 1$ on the boundary, so the maximum modulus principle applied to its inverse shows that there exists $M>1$ such that $|z \, \tau (\ell (z+1/z))| > M$ for all $z \in {\mathbb {C}}_{\le 1-\epsilon }$. The lower bound on $|f_d|$ follows for $d \gg 1$.
- It suffices to prove that $\lim _{\epsilon \rightarrow 0^+} \lim _{d \rightarrow \infty } q P(1/q)^{2/d}$ equals the left endpoint of $I_{attained }$, and likewise at the other end. In fact, (9) implies that $\lim _{d \rightarrow \infty } qP(1/q)^{2/d}$ is a continuous function of $\epsilon \in [0,1]$, so we may simply *substitute* $\epsilon =0$. Then
  $$\begin{aligned} \lim _{d \rightarrow \infty } q P(1/q)^{2/d}&= \lim _{d \rightarrow \infty } q f_d(q^{-1/2})^{2/d} \\&= q \cdot q^{-1/2} q^{-1/2} \, |\tau (\ell (q^{-1/2} + q^{1/2}))| \\&= \tau (q/2 - q^{1/2} + 3/2). \end{aligned}$$
  Similarly, $$\lim _{\epsilon \rightarrow 0^+} \lim _{d \rightarrow \infty } q P(-1/q)^{2/d} = |\tau (-q/2 - q^{1/2} + 1/2)| = \tau (q/2 + q^{1/2} - 1/2)$$.$$\square $$

## Construction of polynomials

We now begin the proof of Theorem 1.1, using the notation introduced in Sect. 3. Let *I* be a closed interval in $I_{attained }$. Let *P*(*z*) be as in Proposition 6.2 and let $d = \deg P$; we may assume that $d \ge 53$.

The polynomial *P* was optimized to have a small value at 1/*q* and large value at $-1/q$. Lemma 7.1 below shows that this makes  small and  large, where *b* is chosen to make $P^b$ of degree close to 2*n*. The polynomial *Q* in Lemma 7.2 interpolates between *P*(*z*) and $P(-z)$ to make  equal a prescribed intermediate value.

### Lemma 7.1

Let $b=b(n)$ and $\ell =\ell (n)$ be functions of *n* tending to $\infty$ such that $\deg P^b = 2n-2\ell$ and $\ell = o(n)$. Thenas $n \rightarrow \infty$. (Recall that , which depends on *n*.)

### Proof

We haveby Lemma 3.5 applied to $P^b$. Taking *n*th roots yields the left endpoint limit, since $\ell \rightarrow \infty$ and $b/n = (2n-2\ell )/(dn) \rightarrow 2/d$. The right endpoint limit follows similarly. $\square$

Choose integers $\ell = \ell (n)$ and $b = b(n)$ such that $\ell = 4 \log _q n + O(1)$ and $bd = 2n - 2\ell$. The statements in the rest of this section will hold if *n* is sufficiently large. Given $m \in {\mathbb {Z}}$ such that $m^{1/n} \in I$, we want to construct an *n*-dimensional, ordinary, geometrically simple, principally polarized abelian variety *A* with $\#A({\mathbb {F}}_q)=m$.

### Lemma 7.2

There exists $Q(z) \in 1+ z {\mathbb {R}}[z]$ of degree $\le d$ such that *Q* is positive on ${\mathbb {R}}$, , and $|Q(w)|^{1/d} \ge q^{-1/4}$ for all $w \in D = {\mathbb {C}}_{\le q^{-1/2}}$.

### Proof

Because *n* is sufficiently large, Proposition 6.2(d) and Lemma 7.1 show that10By the intermediate value theorem, there exists $s \in [-1,1]$ such that the polynomial

$$\begin{aligned} Q(z) \,{:}{=}\,P(sz) \in 1 + z {\mathbb {R}}[z] \end{aligned}$$

satisfies . Thus . Moreover, *Q* is positive on ${\mathbb {R}}$, and $|Q(w)|^{1/d} = |P(sw)|^{1/d} \ge q^{-1/4}$ for all $w \in D$ by Proposition 6.2(b,c). $\square$

In the rest of this section, the implied constant in big-*O* notation may depend on *q*, *L*, *d*, *P*, and *Q*, but not on *n*.

The polynomial $Q^b$ has real coefficients. We could round them to the nearest integer to produce a polynomial $h \in {\mathbb {Z}}[x]$ and adjust the middle coefficients to make ${\widehat{h}}(1) = m$, as in Sect. 4, but it turns out that we cannot guarantee that such an *h* is nonvanishing on *D*, as required for Lemma 3.1. So instead we adjust the coefficients of *Q* (inside the *b*th power) by only *O*(1/*n*) each to make the first *d* coefficients of  integral (and to make them satisfy the congruences in Proposition 5.8), and then, to correct the later coefficients, we add correction polynomials designed to be small on *D*, because as we go along, we need to bound the difference between $Q^b$ and the final *h* to ensure that *h* is still nonvanishing on *D*.

Let us outline the entire construction; then, in a series of lemmas, we will prove that the steps make sense.

### Construction 7.3

Recall the choices of $\ell$ and *b* in the paragraph before Lemma 7.2.

1. Let $Q \in 1 + z {\mathbb {R}}[z]$ be as in Lemma 7.2.
2. Let $g \in ({\mathbb {Z}}/L{\mathbb {Z}})[x]$ be as in Proposition 5.8.
3. Let $$Q_0 = Q$$.
4. For $i=1,\ldots ,d-1$ in turn, let $a_i \in [0,L/b)$ and $Q_i \,{:}{=}\,Q_{i-1} + a_i z^i$ and $h_i \,{:}{=}\,Q_i^b$ be such that ${\widehat{h}}_i^{[2n - i]} \in {\mathbb {Z}}$ and ${\widehat{h}}_i^{[2n - i]} \equiv g^{[2n-i]} \pmod {L}$.
5. Let ${\widetilde{Q}}= Q_{d-1} - c z^d$ and $h_d = {\widetilde{Q}}^b$, where $c \in {\mathbb {R}}$ is chosen so that ${\widehat{h}}_d(1)=m$.
6. Define “correction polynomials” as follows:
  For $$i=d,\ldots ,\ell -1$$, let $$k_i = z^i \, {\widetilde{Q}}(z)^b$$.
  For $i=\ell ,\ldots ,n-1$, let $k_i= z^i \, {\widetilde{Q}}(z)^a$, where $a \in {\mathbb {Z}}_{\ge 0}$ is chosen as large as possible such that $\deg k_i < 2n-i$.
  Define $$k_n = z^n/2$$.
  The definitions are so that ${\widehat{k}}_i$ is monic of degree $2n-i$ for all integers $i \in [d,n]$.
7. For $i=d,\ldots ,n-1$, let $r_i \in [0,L)$ and $s_{i+1} \in {\mathbb {R}}_{\ge 0}$ and $h_{i+1} \,{:}{=}\,h_i + r_i k_i - s_{i+1} k_{i+1}$, where $r_i$ is such that ${\widehat{h}}_{i+1}^{[2n-i]} \in {\mathbb {Z}}$ and ${\widehat{h}}_{i+1}^{[2n-i]} \equiv g^{[2n-i]} \pmod {L}$, and $s_{i+1}$ is such that ${\widehat{h}}_{i+1}(1)=m$.
8. Let *A* be an abelian variety over ${\mathbb {F}}_q$ with $f_A={\widehat{h}}_n$.

### Lemma 7.4

The $a_i$ can be chosen as specified in Step 4, and they are *O*(1/*n*).

### Proof

In Step 4, once $a_1,\ldots ,a_{i-1}$ have been fixed, ${\widehat{h}}_i^{[2n-i]}$ as a function of $a_i$ is a linear polynomial with leading coefficient *b*, so $a_i \in [0,L/b)$ can be found. Then $a_i = O(L/b) = O(1/n)$. $\square$

### Lemma 7.5

- The real number *c* can be chosen as specified in Step 5, and *c* is *O*(1/*n*).
- We have ${\widetilde{Q}}(1)>0$ and ${\widetilde{Q}}(1/q)>0$.
- The values ${\widetilde{Q}}(1)$ and ${\widetilde{Q}}(1/q)$ are *O*(1).

### Proof

- Since $a_i \ge 0$, we have $Q_{d-1} \ge \cdots \ge Q_0 = Q > 0$ on ${\mathbb {R}}_{\ge 0}$, so 11 Let
  $$\begin{aligned} c' \,{:}{=}\,q^{d-1} a_1 + q^{d-2} a_2 + \ldots + q a_{d-1}. \end{aligned}$$
  Let $$R = Q_{d-1} - c' z^d$$. Then
  $$\begin{aligned} R(1) = Q_{d-1}(1) - c' = Q(1) - (q^{d-1} - 1) a_1 - \cdots - (1-1) a_{d-1} \in (0,Q(1)], \end{aligned}$$
  for large *n*, by Lemma 7.4, and
  $$\begin{aligned} R(1/q)&= Q_{d-1}(1/q) - c'/q^d \\&= (Q(1/q) + a_1 q^{-1} + \cdots +a_{d-1} q^{-(d-1)}) - (a_1 q^{-1} + \cdots +a_{d-1} q^{-(d-1)}) \\&= Q(1/q) > 0, \end{aligned}$$
  so 12 By (11) and (12) and the intermediate value theorem, there exists $c \in [0,c']$ such that . Moreover, $c=O(c') = O((d-1) q^{d-1} (1/n)) = O(1/n)$.
- We have ${\widetilde{Q}}(1) \ge R(1) > 0$ and ${\widetilde{Q}}(1/q) \ge R(1/q) > 0$.
- For $w \in \{1,1/q\}$, we have ${\widetilde{Q}}(w) = Q(w) + O(1/n)$, and $Q(w) \in P([-1,1])$, an interval independent of *n*. $\square$

Lemmas 7.6 through 7.9 show that ${\widetilde{Q}}^b$ is large enough on *D* and the corrections are small enough that $h_n$ is nonvanishing on *D*.

### Lemma 7.6

We have $|{\widetilde{Q}}(w)| \ge q^{-d/4} - O(1/n)$ for every $w \in D$.

### Proof

By Lemma 7.2, $|Q(w)| \ge q^{-d/4}$, and $|{\widetilde{Q}}(w)|$ differs from |*Q*(*w*)| by at most $|a_1 w + \cdots + a_{d-1} w^{d-1} - c w^d| = O(1/n)$, by Lemmas 7.4 and 7.5. $\square$

### Lemma 7.7

We have $k_i(1)>0$ and $k_i(1/q)>0$.

### Proof

These follow from Lemma 7.5(b). $\square$

### Lemma 7.8

The $r_i \in [0,L)$ and $s_{i+1}$ can be chosen as specified in Step 7, and $s_{i+1}$ is *O*(1). For $i=d,\ldots ,\ell -2$, we have the more precise bound $s_{i+1} \in [0,qL]$.

### Proof

This is similar to the proof of Lemma 7.5. The value ${\widehat{h}}_{i+1}^{[2n-i]}$ is $r_i$ plus terms that have already been fixed, so there is a unique choice $r_i \in [0,L)$ such that ${\widehat{h}}_{i+1}^{[2n-i]} \in {\mathbb {Z}}$ and ${\widehat{h}}_{i+1}^{[2n-i]} \equiv g^{[2n-i]} \pmod {L}$.

We seek $s_{i+1}$ making the value ${\widehat{h}}_{i+1}(1) = m + r_i \, {\widehat{k}}_i(1) - s_{i+1} \, {\widehat{k}}_{i+1}(1)$ equal to *m*. By Lemma 7.7,

13 $$\begin{aligned} m + r_i \, {\widehat{k}}_i(1) \ge m. \end{aligned}$$

Let $V=k_i/k_{i+1}$ and $v = \max \{V(1),V(1/q)\}$. By Lemma 7.7, ${\widehat{k}}_i(1) \le v \, {\widehat{k}}_{i+1}(1)$, so

14 $$\begin{aligned} m + r_i \, {\widehat{k}}_i(1) - v r_i \, {\widehat{k}}_{i+1}(1) \le m. \end{aligned}$$

Now (13), (14), and the intermediate value theorem yield $s_{i+1} \in [0,v r_i] \subseteq [0, v L]$ making ${\widehat{h}}_{i+1}(1)=m$.

To bound $s_{i+1}$, we need to bound *v*. The function *V* is 1/*z*, ${\widetilde{Q}}(z)/z$, or 2/*z*; accordingly, *v* is *q*, *O*(1), or 2*q*, with the middle case following from Lemma 7.5(b,c). In every case, $v = O(1)$, so $s_{i+1} = O(1)$. If $i \in [d,\ell -1)$, then $V=1/z$, so $v=q$, so $s_{i+1} \in [0,qL]$. $\square$

### Lemma 7.9

The polynomial $h_n$ is nonvanishing on *D*.

### Proof

By construction,

$$\begin{aligned} h_n = {\widetilde{Q}}^b + \sum _{i=d}^{n-1} (r_i k_i - s_{i+1} k_{i+1}), \end{aligned}$$

so it suffices to prove that

15 $$\begin{aligned} \sum _{i=d}^{n-1} \left| \frac{r_i k_i}{{\widetilde{Q}}^b} \right| + \sum _{i=d+1}^{n} \left| \frac{s_i k_i}{{\widetilde{Q}}^b} \right| < 1 \end{aligned}$$

on *D*. We claim that

16 $$\begin{aligned} \left| \frac{k_i}{{\widetilde{Q}}^b} \right| \le {\left\{ \begin{array}{ll} q^{-i/2} &  if i \in [d,\ell ), \\ O(n^{-2}) &  if i \in [\ell ,n], \end{array}\right. } \end{aligned}$$

on *D*. The case $i \in [d,\ell )$ follows since $k_i/{\widetilde{Q}}^b=z^i$. In particular, for $i \in [\ell -d/2,\ell )$, we have $|k_i/{\widetilde{Q}}^b| \le q^{-(\ell -d/2)/2} = O(q^{-\ell /2}) = O(n^{-2})$. From then on, changing *i* to $i+d/2$ multiplies $|k_i/{\widetilde{Q}}^b|$ by $|z^{d/2}/{\widetilde{Q}}| \le q^{-d/4} / (q^{-d/4} - O(1/n)) = 1 + O(1/n)$ by Lemma 7.6 (or, at the last step with $i+d/2=n$, by $|(z^n/2)/z^i| = |z^{d/2}/2| \le 1$); this happens fewer than *n* times, and $(1+O(1/n))^n =O(1)$, so (16) for $i \in [\ell ,n]$ follows.

By Lemma 7.8 and (16), the left hand side of (15) is at most

$$\begin{aligned}  &   \sum _{i=d}^{\ell -1} L q^{-i/2} + \sum _{i=\ell }^{n-1} L \, O(n^{-2}) + \sum _{i=d+1}^{\ell -1} qL \, q^{-i/2} + \sum _{i=\ell }^n O(1) \, O(n^{-2})\\    &   \quad \le \frac{2 L q^{-(d-1)/2}}{1-q^{-1/2}} + O(1/n) < 1 \end{aligned}$$

if *n* is large, since $L < q^{23}$ and $d \ge 53$. $\square$

### Lemma 7.10

The polynomial ${\widehat{h}}_n$ is monic of degree 2*n*. Also, ${\widehat{h}}_n \in {\mathbb {Z}}[x]$ and ${\widehat{h}}_n \equiv g \pmod {L}$.

### Proof

In Steps 4 and 7, adjusting $h_i$ to produce $h_{i+1}$ does not change the coefficients of $z^{2n}$, $z^{2n-1}$, ..., $z^{2n-i}$ in ${\widehat{h}}_i$, which are integers congruent modulo *L* to the corresponding coefficients of *g*; by *q*-symmetry, the same holds for the coefficients of 1, *z*, ..., $z^i$. Thus ${\widehat{h}}_n$ is monic and has integer coefficients congruent to the coefficients of *g*, except perhaps the coefficient of $z^n$; actually it holds for this coefficient too since ${\widehat{h}}_n(1)$ is an integer (namely, *m*) and ${\widehat{h}}_n(1) = m \equiv g(1) \pmod {L}$. $\square$

### End of proof of Theorem 1.1

 

- The polynomial ${\widehat{h}}_n$ is monic of degree 2*n*, with integer coefficients, by Lemma 7.10.
- All complex roots of ${\widehat{h}}_n$ have absolute value $q^{1/2}$, by Lemmas 7.9 and 3.1.
- The characteristic *p* does not divide ${\widehat{h}}_n^{[n]}$, because by Lemma 7.10, ${\widehat{h}}_n^{[n]}$ is congruent modulo *L* to $g^{[n]}$, which is nonzero modulo *p*, and $p \mid L$, by construction of *g*.

By Theorem 2.1, there exists an ordinary *n*-dimensional abelian variety *A* over ${\mathbb {F}}_q$ with $f_A = {\widehat{h}}_n$. Then $\#A({\mathbb {F}}_q)=f_A(1) = {\widehat{h}}_n(1) = m$. By Proposition 5.8, *A* is geometrically simple, and principally polarized after replacing *A* by an isogenous abelian variety. $\square$

## Large *q* limit

In this section, we prove Theorem 1.7, which for fixed *n* and large *q* determines the largest subinterval of the Hasse–Weil interval in which all integers are realizable as $\#A({\mathbb {F}}_q)$ for an *n*-dimensional abelian variety *A* over ${\mathbb {F}}_q$. Throughout this section, in big-*O* notation, the implied constant depends on *n* but not *q*.

First let us explain the idea. For any *n*-dimensional abelian variety *A* over ${\mathbb {F}}_q$, we have $f_A(x) = x^n \, G(x+q/x)$ for some polynomial

17 $$\begin{aligned} G(x) = x^n + c_1 x^{n-1} + c_2 x^{n-2} + \cdots + c_n \in {\mathbb {Z}}[x] \end{aligned}$$

all of whose roots lie in $[-2q^{1/2},2q^{1/2}]$. Then $c_i = O(q^{i/2})$, and

$$\begin{aligned} \#A({\mathbb {F}}_q) = f_A(1) = G(q+1) = (q+1)^n + c_1 (q+1)^{n-1} + c_2 (q+1)^{n-2} + \cdots + c_n. \end{aligned}$$

**Fig. 1:**
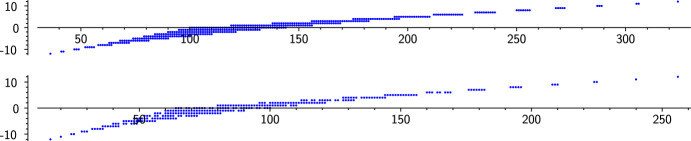
For $q=11$ and $q=9$, respectively, the graph shows all the points $(\#A({\mathbb {F}}_q),c_1)$, where *A* ranges over abelian surfaces over ${\mathbb {F}}_q$, and $c_1 = G^{[n-1]} = f_A^{[2n-1]}$ with $n=2$; see (17)

For each integer $c_1$ in the possible range $[-2n q^{1/2},2n q^{1/2}]$, let $I_{c_1}$ be the smallest interval containing the possible values of $c_2 (q+1)^{n-2} + \cdots + c_n$; then we prove that the ranges for $c_2$, ..., $c_n$ are large enough that all integers in $I_{c_1}$ are realized, possibly ignoring a negligible fraction of the interval at the ends. The interval $I_{c_1}$ has width $O(q^{n-1})$ and does not change much when $c_1$ is incremented by 1 — its endpoints move by $o(q^{n-1})$. The big-*O* constant matters: for $c_1$ close to the extremes of its range (with $|c_1|$ greater than about $\left( 2n -\sqrt{\frac{2n}{n-1}} \right) q^{1/2}$), it turns out that $I_{c_1}$ has length significantly less than $q^{n-1}$, so that there is a gap between the intervals $(q+1)^n + c_1(q+1)^{n-1} + I_{c_1}$ and $(q+1)^n + (c_1+1)(q+1)^{n-1} + I_{c_1+1}$, a gap in which $\#A({\mathbb {F}}_q)$ cannot lie; see Lemma 8.2. On the other hand, for the $c_1$ towards the middle of the range, $I_{c_1}$ has width significantly greater than $q^{n-1}$, so the intervals $(q+1)^n + c_1(q+1)^{n-1} + I_{c_1}$ overlap to cover a large interval in the middle of the Hasse–Weil interval. Figure 1 shows these overlapping intervals when $n=2$ and $q \in \{11,9\}$; for the non-prime 9, there is an additional phenomenon explained in Remark 8.5.

As the previous paragraph indicates, the coefficients of $x^{n-1}$ and $x^{n-2}$ are what matter most. After using the normalization $g(x) \,{:}{=}\,q^{-n/2} G(q^{1/2} x)$, we are led to study

$$\begin{aligned} {\mathcal {G}}&\,{:}{=}\,\{ \, g \in {\mathbb {R}}[x] : { g} is monic of degree { n} with all roots in [-2,2] \, \}\\ {\mathcal {S}}&\,{:}{=}\,\{ \, (g^{[n-1]},g^{[n-2]}) \in {\mathbb {R}}^2 : g \in {\mathcal {G}}\, \}, \end{aligned}$$

equipped with the Euclidean topology. Let $\lambda _1 = 2n - \sqrt{\tfrac{2n}{n-1}}$ and $\lambda _2 = 2n - \sqrt{\tfrac{4n}{n-1}}$.

### Lemma 8.1

If $n \ge 2$, then there exist continuous functions $B_{\min },B_{\max } :[-2n,2n] \rightarrow {\mathbb {R}}$ such that

- We have $${\mathcal {S}}= \{\, (a,b) \in [-2n,2n] \times {\mathbb {R}}: B_{\min }(a) \le b \le B_{\max }(a) \, \}$$.
- The difference $B_{diff }(a) \,{:}{=}\,B_{\max }(a) - B_{\min }(a)$ is
  nonnegative on $[-2n,2n]$, positive on $(-2n,2n)$,
  less than 1 if $\lambda _1 < |a| \le 2n$, greater than 1 if $|a|<\lambda _1$,
  less than 2 if $\lambda _2 < |a| \le 2n$, and greater than 2 if $|a|<\lambda _2$.
- There exists a compact subset ${\mathcal {G}}_0 \subset {\mathcal {G}}$ surjecting onto ${\mathcal {S}}$ such that any $g \in {\mathcal {G}}_0$ mapping into the interior of ${\mathcal {S}}$ has distinct roots in $(-2,2)$.

### Proof

If $g=\prod _{i=1}^n (x-r_i)$, then $(g^{[n-1]},g^{[n-2]}) = (-\sum r_i,\sum _{i<j} r_i r_j)$. Given $a \in [-2n,2n]$, let

$$\begin{aligned} {\mathcal {C}}_a = \{ (r_1,\ldots ,r_n) \in [-2,2]^n: \textstyle \sum r_i =-a\}. \end{aligned}$$

Since ${\mathcal {C}}_a$ is compact and connected, (a) holds with $B_{\min }$ and $B_{\max }$ being the minimum and maximum of $\sum _{i<j} r_i r_j$ on ${\mathcal {C}}_a$. If any two of the $r_i$ are different, then we can increase $\sum _{i<j} r_i r_j$ by replacing both by their average; thus the maximum occurs when the $r_i$ are all equal, so $$B_{\max }(a) = \left( {\begin{array}{c}n\\ 2\end{array}}\right) (a/n)^2$$. If there are two $r_i$ in $(-2,2)$, then we can decrease $\sum _{i<j} r_i r_j$ by subtracting $\epsilon$ from the smaller and adding $\epsilon$ to the larger, for some $\epsilon >0$; thus the minimum occurs when all but one $r_i$ are at $\pm 2$. Given *a*, there is at most one such $(r_1,\ldots ,r_n)$ with $\sum r_i=-a$ up to permuting coordinates: as *a* increases, the roots move linearly from 2 to $-2$ one at a time. So $B_{\min }$ is the piecewise-linear continuous function such that for each $k \in \{0,\ldots ,n-1\}$,

$$\begin{aligned}  &   B_{\min }(a) = (4k-2n+2) a -8 k^2+8 k (n-1)-2 (n-1) n\\  &   \quad for a \in [4k-2n,4k-2n+4] . \end{aligned}$$

The minimum value of $B_{diff }$ on $[4k-2n,4k-2n+4]$ is

$$\begin{aligned} B_{diff }(4k-2n + 4k/(n-1)) = 8k(n-1-k)/(n-1), \end{aligned}$$

which for $k \in \{1,\ldots ,n-2\}$ is at least $8(n-2)/(n-1) \ge 4$. On the other hand, for $t \in [0,4]$, we have $B_{diff }(2n-t) = B_{diff }(-2n+t) = \frac{n-1}{2n} t^2$. The claims in (b) follow.

Given *a*, let $\prod _{i=1}^n (x-r_i)$ and $\prod _{i=1}^n (x-r_i'')$ be the polynomials realizing $B_{\min }(a)$ and $B_{\max }(a)$, each with roots listed in increasing order. (So all but one $r_i$ are $\pm 2$, and $r_i''=-a/n$ for all *i*.) Let $\epsilon \ge 0$ be the distance from $-a/n$ to the boundary of $[-2,2]$, and let $r_1',\ldots ,r_n'$ be an arithmetic progression with $r_1' = -a/n-\epsilon /2$ and $r_n'=-a/n+\epsilon /2$. For each $s \in [0,1]$, consider the monic degree *n* polynomial whose roots are $(1-s) r_i + s r_i'$ for $i=1,\ldots ,n$ and the analogous polynomial with roots $(1-s) r_i' + s r_i''$. These depend continuously on $(a,s) \in [-2n,2n] \times [0,1]$, so the set of all such polynomials is a compact subset ${\mathcal {G}}_0$ of ${\mathcal {G}}$. For fixed *a*, the coefficients of $x^{n-2}$ in these polynomials vary continuously from $B_{\min }(a)$ to $B_{\max }(a)$, so ${\mathcal {G}}_0 \rightarrow {\mathcal {S}}$ is surjective. Finally, by construction, all polynomials in ${\mathcal {G}}_0$ except for the ones realizing $B_{\min }(a)$ and $B_{\max }(a)$ have distinct roots in $(-2,2)$. $\square$

### Lemma 8.2

Suppose $n \ge 2$. For $\lambda \in {\mathbb {R}}$ satisfying $\lambda _1< |\lambda | < 2n$, there exists $\epsilon >0$ such that if *q* is sufficiently large and $r = \lfloor \lambda q^{1/2} \rfloor$, then the interval

18 $$\begin{aligned}  &   \bigl [ (q+1)^n + r (q+1)^{n-1} + (B_{\max }(\lambda ) + \epsilon ) q^{n-1},\nonumber \\  &   (q+1)^n + (r+1) (q+1)^{n-1} + (B_{\min }(\lambda ) - \epsilon ) q^{n-1} \bigr ] \end{aligned}$$

has width $>1$ and does not contain $\#A({\mathbb {F}}_q)$ for any *n*-dimensional abelian variety *A* over ${\mathbb {F}}_q$.

### Proof

By Lemma 8.1(b), $B_{diff }(\lambda ) < 1$. Choose $\epsilon >0$ such that $B_{diff }(\lambda ) < 1-2\epsilon$. Then the width of the interval (18) is $(q+1)^{n-1} - (B_{diff }(\lambda ) + 2 \epsilon ) q^{n-1} > 1$.

Let *A* be an *n*-dimensional abelian variety over ${\mathbb {F}}_q$. Then $f_A(x) = x^n \, G(x+q/x)$ for some $G(x) = x^n + c_1 x^{n-1} + \cdots + c_n \in {\mathbb {Z}}[x]$ with all roots in $[-2q^{1/2},2q^{1/2}]$. We have $c_i = O(q^{i/2})$ and $(a,b) \,{:}{=}\,(q^{-1/2} c_1,q^{-1} c_2) \in {\mathcal {S}}$. Now

19 $$\begin{aligned} \#A({\mathbb {F}}_q) = f_A(1) = G(q+1) = (q+1)^n + c_1 (q+1)^{n-1} + b q^{n-1} + O(q^{n-3/2}).\nonumber \\ \end{aligned}$$

Since $b = O(1)$, if $\#A({\mathbb {F}}_q)$ lies in the interval (18), then $c_1 = r + O(1)$, so $a = q^{-1/2} c_1 = \lambda + O(q^{-1/2})$. Then

$$\begin{aligned} b \in [B_{\min }(a),B_{\max }(a)] \subset [B_{\min }(\lambda )-\epsilon /2,B_{\max }(\lambda )+\epsilon /2] \end{aligned}$$

by continuity, if *q* is large enough. If $c_1 \le r$, then the right side of (19) is too small to lie in (18). If $c_1 \ge r+1$, then it is too large. $\square$

### Lemma 8.3

Suppose that $n \ge 3$ and $\lambda \in {\mathbb {R}}$ satisfies $0< \lambda < \lambda _1$. Then for sufficiently large *q*, every integer in

20 $$\begin{aligned} \bigl [ q^n - \lambda q^{n-1/2} , q^n + \lambda q^{n-1/2} \bigr ] \end{aligned}$$

is $\#A({\mathbb {F}}_q)$ for some *n*-dimensional abelian variety *A* over ${\mathbb {F}}_q$.

### Proof

By Lemma 8.1(b), $B_{diff } >1$ on $[-\lambda ,\lambda ]$. Choose $\epsilon >0$ so that $B_{diff } > 1 + 2\epsilon$ on $[-\lambda ,\lambda ]$. Let

$$\begin{aligned} {\mathcal {S}}_\epsilon = \{\, (a,b) \in [-2n,2n] \times {\mathbb {R}}: B_{\min }(a) + \epsilon \le b \le B_{\max }(a) - \epsilon \, \}. \end{aligned}$$

Then ${\mathcal {S}}_\epsilon$ is a compact subset of the interior of ${\mathcal {S}}$. Let ${\mathcal {G}}_\epsilon$ be the inverse image of ${\mathcal {S}}_\epsilon$ under ${\mathcal {G}}_0 \twoheadrightarrow {\mathcal {S}}$. By Lemma 8.1(c), ${\mathcal {G}}_\epsilon$ is compact and consists of polynomials with distinct real roots in $(-2,2)$, so we can choose $\delta >0$ such that any polynomial whose coefficients are within $\delta$ of some $g \in {\mathcal {G}}_\epsilon$ again has distinct real roots in $(-2,2)$.

Suppose that *m* is an integer in $[ q^n - \lambda q^{n-1/2} , q^n + \lambda q^{n-1/2} ]$. The rest of the proof relies on the following construction.

### Construction 8.4

 

1. Let $a \in [-\lambda ,\lambda ]$ be such that $m = q^n + a q^{n-1/2}$.
2. Write $m = (q+1)^n + (c_1 + b) (q+1)^{n-1}$ with $c_1 \in {\mathbb {Z}}$ and $b \in [ B_{\min }(a) + \epsilon , B_{\max }(a) -\epsilon ]$ (possible since $[ B_{\min }(a) + \epsilon , B_{\max }(a) -\epsilon ]$ has length $>1$). Then $(a,b) \in {\mathcal {S}}_\epsilon$.
3. Choose $g \in {\mathcal {G}}_\epsilon$ mapping to (*a*, *b*).
4. Let $$G(x) = q^{n/2} \, g(q^{-1/2} x) = x^n + q^{1/2} a x^{n-1} + q b x^{n-2} + \cdots \in {\mathbb {R}}[x]$$.
5. Let $G_1$ be the same as *G* except with the coefficient of $x^{n-1}$ changed to $c_1$.
6. For $i=2,\ldots ,n$, let $G_i$ be the same as $G_{i-1}$ except with the coefficient of $x^{n-i}$ changed to the integer $c_i$ that makes $G_i(q+1)-m \in [0,(q+1)^{n-i})$.
7. Let $G_{final } = G_n + s(x-(q+1))$, where $s \in \{0,1\}$ is chosen so that $p \not \mid G_{final }^{[0]}$.

*Continuation of proof of Lemma* 8.3. We now bound the coefficients of $G_{final } - G$ in order to prove that for *q* large enough, the roots of $G_{final }$ are still distinct and all in $[-2q^{1/2},2q^{1/2}]$. Since (*a*, *b*) lies in ${\mathcal {S}}_\epsilon$, which is compact, *b* is *O*(1). By Steps 1 and 2,

21 $$\begin{aligned} q^n + a q^{n-1/2}&= m = (q+1)^n + (c_1+b)(q+1)^{n-1} = q^n + c_1 q^{n-1} + O(q^{n-1}), \nonumber \\ c_1&= q^{1/2} a + O(1). \end{aligned}$$

Now

$$\begin{aligned} G_1(q+1)&= (q+1)^n + c_1 (q+1)^{n-1} + qb (q+1)^{n-2}\\&\quad + O(q^{3/2}) (q+1)^{n-3} + \cdots + O(q^{n/2}) (q+1)^0 \\&= (q+1)^n + (c_1 + b)(q+1)^{n-1} + O(q^{n-3/2}) \\&= m + O(q^{n-3/2}), \end{aligned}$$

so

22 $$\begin{aligned} c_2 - G^{[n-2]} = O(q^{n-3/2}) / (q+1)^{n-2} = O(q^{1/2}). \end{aligned}$$

Similarly, for $i=3,\ldots ,n$, we have

23 $$\begin{aligned} c_i - G^{[n-i]} = O((q+1)^{n-(i-1)})/(q+1)^{n-i} = O(q). \end{aligned}$$

Equations (21), (22), and (23) imply that

$$\begin{aligned} G_n^{[n-i]} - G^{[n-i]} = O(q^{(i-1)/2}) \end{aligned}$$

for all $i \ge 1$. Since $n \ge 3$, the same holds with $G_n$ replaced by $G_{final }$. Thus the coefficients of $g_{final }(x) = q^{-n/2} \, G_{final }(q^{1/2} x)$ are within $O(q^{-1/2}) < \delta$ of the corresponding coefficients of *g* if *q* is sufficiently large, so $g_{final }$ has all its roots in $[-2,2]$. Thus $G_{final }$ has all its roots in $[-2q^{1/2},2q^{1/2}]$. By construction, $G_{final } \in {\mathbb {Z}}[x]$. Also $G_{final }(q+1) - m = G_n(q+1) - m \in [0,1)$, so $G_{final }(q+1)=m$.

Let $f(x)=x^n \, G_{final }(x+q/x) \in {\mathbb {Z}}[x]$. We have $f^{[n]} \equiv G_{final }^{[0]} \not \equiv 0 \pmod {p}$. By Theorem 2.1, $f=f_A$ for some *n*-dimensional ordinary abelian variety over ${\mathbb {F}}_q$. Finally, $\#A({\mathbb {F}}_q)=f(1)=G_{final }(q+1)=m$. $\square$

### Proof of Theorem 1.7

Lemma 8.3 shows that all integers in $[ q^n - \lambda q^{n-1/2}, q^n + \lambda q^{n-1/2} ]$ are realizable for $\lambda$ that can approach $\lambda _1$ from below as $q \rightarrow \infty$. Lemma 8.2 shows, on the other hand, that for any $\mu$ with $|\mu | > \lambda _1$, there are unrealizable integers within $O(q^{n-1})$ of $(q+1)^n + \mu q^{n-1/2}$ if *q* is sufficiently large. These imply Theorem 1.7. $\square$

### Remark 8.5

Suppose $n=2$. Theorem 1.7 holds without change if *q* tends to $\infty$ through primes only: the proof of Lemma 8.3 works if we omit Step 7, because of the last sentence of Remark 2.2.

On the other hand, if *q* tends to $\infty$ through non-prime prime powers, then Theorem 1.7 holds with $\lambda _1$ replaced by the smaller value $\lambda _2 = 4-2\sqrt{2}$, as we now explain. In Lemma 8.3, if $0< \lambda < \lambda _2$, then $B_{diff } > 2$ on $[-\lambda , \lambda ]$, so there are at least *two* consecutive integer possibilities for $c_1$, and at least one of them will lead to a polynomial *f* for which (c) in Theorem 2.1 holds. Meanwhile, in Lemma 8.2, if $\lambda _2< |\mu | < 2n$, so that $B_{diff }(\mu ) < 2$, then there exists $\epsilon >0$ such that if *q* is sufficiently large, and *r* is the multiple of *p* nearest $\mu q^{1/2}$, then any integer of the form $m=(q+1)^2 + r (q+1) +c_2$ in

$$\begin{aligned}  &   \bigl [ (q+1)^2 + (r-1) (q+1) + (B_{\max }(\mu ) + \epsilon ) q ,\\  &   (q+1)^2 + (r+1) (q+1) + (B_{\min }(\mu ) - \epsilon ) q \bigr ] \end{aligned}$$

with $p \mid c_2$ and $p^2 \not \mid c_2$ is not $\#A({\mathbb {F}}_q)$ for any abelian surface *A* over ${\mathbb {F}}_q$, because the only monic quadratic polynomial $G(x) \in {\mathbb {Z}}[x]$ with roots in $[-2q^{1/2},2q^{1/2}]$ such that $G(q+1)=m$ is $x^2+rx+c_2$, which is Eisenstein at *p*, which implies that the polynomial $f(x) \,{:}{=}\,x^2 \, G(x+q/x)$ fails condition (c_1_) in Remark 2.2.

## Effective bounds

Given *q* and *n*, we have given three ways to construct polynomials that realize a large interval of integers as $\#A({\mathbb {F}}_q)$ for *A* of dimension *n* over ${\mathbb {F}}_q$:

- Sect. 4 gave a quick construction that realized intervals wide enough to cover all sufficiently large integers as *n* varies, but not wide enough to be asymptotically close to optimal.
- Sect. 7 gave a more subtle construction that gave a much wider interval, but it is too complicated to analyze explicitly to make all the big-*O* constants explicit.
- Sect. 8 gave a method that again is asymptotically good, but only when *q* is large compared to *n*.

In this section, we present a *fourth* construction that, while not asymptotically as good as the construction of Sect. 7, realizes a wide interval for any *q* and sufficiently large *n*, and is still simple enough to analyze fully.

Given *q*, $$n \ge 2$$, and an integer *m* in $$[q^{n-1/2},q^{n+1/2})$$, the plan is to find a power series $$j(z) \in z{\mathbb {R}}[[z]]$$ such that $$j(1/q) = \log (m/q^n)$$ and $$\exp (j(z)) \in {\mathbb {Z}}[[z]]$$; then we truncate $$\exp (j(z))$$ to a degree *n* polynomial and adjust the coefficients of $$x^{n-1}$$ and $$x^n$$ to produce a polynomial *h*(*z*) such that $${\widehat{h}}(1)=m$$ and $$p \not \mid {\widehat{h}}^{[n]}$$. This should work well, since $$\exp (j(z))$$ is automatically nonvanishing on *D*, and if its coefficients are not too large, then the nonvanishing should persist after truncating and adjusting.

### Construction 9.1

 

1. For $i=1,2,\ldots ,n-1$ in turn, let $c_i$ be the real number such that
  $$\begin{aligned} \log (m/q^n) - c_1 q^{-1} - \cdots - c_i q^{-i} \in [-q^{-i}/2,q^{-i}/2) \end{aligned}$$
  and such that the coefficient of $z^i$ in the power series $\exp (c_1 z + \cdots + c_i z^i)$ is an integer; for the existence and uniqueness of $c_i$, see the proof of Lemma 9.2.
2. Let $c_n \in {\mathbb {R}}$ be such that $\log (m/q^n) - c_1 q^{-1} - \cdots - c_n q^{-n} = 0$.
3. Let $h_0(z) \in {\mathbb {R}}[z]$ be the degree *n* Taylor polynomial of $\exp (c_1 z + \cdots + c_n z^n)$.
4. Let $h_1(z) = h_0(z) + k z^n/2$, where $k \in {\mathbb {R}}$ is chosen to make ${\widehat{h}}_1(1)=m$.
5. Let *h* be $h_1$ or $h_1 + z^{n-1} - ((q+1)/2) z^n$, whichever makes $p \not \mid {\widehat{h}}^{[n]}$.
6. Let *A* be an abelian variety with $f_A={\widehat{h}}$, if one exists. (If *h* is nonvanishing on *D*, then such an *A* is guaranteed to exist and ${\widehat{h}}$ is squarefree by Remark 3.3.)

Let $$s = \lfloor \frac{1}{2} q \log q + \frac{1}{2} \rfloor $$.

### Lemma 9.2

We have $|c_1| \le s$ and $|c_i| \le (q+1)/2$ for $i=2,\ldots ,n$.

### Proof

Since $m \in [q^{n-1/2},q^{n+1/2})$, we have $\log (m/q^n) \in [-\frac{1}{2} \log q, \frac{1}{2} \log q)$, and Step 1 says that $c_1$ is the integer in the interval $q \log (m/q^n) + (-\frac{1}{2},\frac{1}{2}]$, so $|c_1| \le s$.

For $i \le n-1$, let $\epsilon _i =\log (m/q^n) - c_1 q^{-1} - \cdots - c_i q^{-i}$, so $\epsilon _i = \epsilon _{i-1} - c_i q^{-i}$; then $\epsilon _{i-1} \in [-q^{-(i-1)}/2,q^{-(i-1)}/2)$, so the condition $\epsilon _i \in [-q^{-i}/2,q^{-i}/2)$ in Step 1 constrains $c_i$ to a half-open interval of length 1 contained in $[-(q+1)/2, (q+1)/2]$, while the integer coefficient condition in Step 1 constrains $c_i$ to a coset of ${\mathbb {Z}}$ in ${\mathbb {R}}$; thus a unique $c_i$ exists, and $|c_i| \le (q+1)/2$. Finally, $c_n = q^n \epsilon _{n-1} \in q^n[-q^{-(n-1)}/2,q^{-(n-1)}/2) = [-q/2,q/2)$. $\square$

Let $$j(z) = c_1 z + \cdots + c_n z^n$$. Let

$$\begin{aligned} J(z) \,{:}{=}\,\exp \left( sz + \frac{q+1}{2} \frac{z^2}{1-z} \right) = J_{\le n}(z) + J_{>n}(z) \quad \in {\mathbb {R}}_{\ge 0}[[z]], \end{aligned}$$

where $J_{\le n}$ is the degree *n* Taylor polynomial, and $J_{>n}$ is the remainder power series consisting of terms of degree $>n$. By Lemma 9.2, $|(\exp j(z))^{[i]}| \le J^{[i]}$.

### Proposition 9.3

Let *q* be a prime power. For $n \ge 2$ and $m \in [q^{n-1/2},q^{n+1/2})$, if

24 $$\begin{aligned} J_{>n}(q^{-1/2}) + \frac{q^{n/2}}{2} J_{>n}(q^{-1}) + \frac{q^{-n/2}}{2} J_{\le n}(1) + \frac{(q^{1/2}+1)^2}{2} q^{-n/2} < \frac{1}{J(q^{-1/2})}, \end{aligned}$$

then Construction 9.1 produces an ordinary *n*-dimensional *A* over ${\mathbb {F}}_q$ with $\#A({\mathbb {F}}_q)=m$.

### Proof

By Step 2, $\exp j(q^{-1}) = m/q^n$, so

$$\begin{aligned} |m - q^n h_0(q^{-1}) |= q^n |\exp j(q^{-1}) - h_0(q^{-1}) |\le q^n \, J_{>n}(q^{-1}), \\ |h_0(1) |\le J_{\le n}(1), \\ |k| = |{\widehat{h}}(1)- {\widehat{h}}_0(1) |\le |m - q^n h_0(q^{-1}) - h_0(1) |\le q^n \, J_{>n}(q^{-1}) + J_{\le n}(1). \end{aligned}$$

On *D*,

$$\begin{aligned} |\exp j(z) |&= \exp ({{\,\textrm{Re}\,}}j(z))\\&\ge \exp \left( -s q^{-1/2} - \frac{q+1}{2} q^{-2/2} - \cdots - \frac{q+1}{2} q^{-n/2} \right) \ge \frac{1}{J(q^{-1/2})}, \\ |h_0(z) |&\ge |\exp j(z) |- J_{>n}(q^{-1/2}), \\ |h_1(z) |&\ge |h_0(z) |- \frac{k}{2} q^{-n/2}, \\ |h(z) |&\ge |h_1(z) |- q^{-(n-1)/2} - \frac{q+1}{2} q^{-n/2} = |h_1(z) |- \frac{(q^{1/2}+1)^2}{2} q^{-n/2}. \end{aligned}$$

Combining the previous five inequalities yields

$$\begin{aligned} |h(z)|&\ge \frac{1}{J(q^{-1/2})} - J_{>n}(q^{-1/2}) - \frac{q^{n/2}}{2} J_{>n}(q^{-1}) - \frac{q^{-n/2}}{2} J_{\le n}(1) - \frac{(q^{1/2}+1)^2}{2} q^{-n/2}, \end{aligned}$$

so (24) implies that *h* is nonvanishing on *D*. Theorem 2.1 produces *A*. $\square$

The following weaker statement has the advantage that if any hypothesis holds for one *n*, it clearly holds for all larger *n* (since *J* has nonnegative coefficients):

### Corollary 9.4

Let *q* be a prime power. For $n \ge 2$ and $m \in [q^{n-1/2},q^{n+1/2})$, if any of

25 $$\begin{aligned}  &   (1+q^{-1/2}/2) J_{>n}(q^{-1/2}) + \tfrac{1}{2} \left( \tfrac{4}{3} q^{-1/2} \right) ^n J(\tfrac{3}{4}) + \frac{(q^{1/2}+1)^2}{2} q^{-n/2} < \frac{1}{J(q^{-1/2})}, \nonumber \\\end{aligned}$$

26 $$\begin{aligned}  &   q \ge 7 \quad and \quad 2^{n-1} > J(q^{-1/2}) J(2q^{-1/2}), \quad or \end{aligned}$$

27 $$\begin{aligned}  &   q \ge 16 \quad and \quad n > 3 q^{1/2} \log q - 1/2 \end{aligned}$$

hold, then Construction 9.1 produces an ordinary *n*-dimensional *A* over ${\mathbb {F}}_q$ with $\#A({\mathbb {F}}_q)=m$.

### Proof

In (24), $J_{>n}(q^{-1}) \le q^{-(n+1)/2} J_{>n}(q^{-1/2})$ (this holds termwise for any power series with nonnegative coefficients). Similarly $J_{\le n}(1) \le (\frac{4}{3})^n J_{\le n}(3/4) \le (\frac{4}{3})^n J(3/4)$. Hence the left side of (24) is at most the left side of (25). Thus, if (25) holds, Proposition 9.3 applies.

Now suppose that $q \ge 7$ and $2^{n-1} > J(q^{-1/2} )J(2 q^{-1/2})$. First,

$$\begin{aligned} 2^{n-1} > J(q^{-1/2}) J(2q^{-1/2}) \ge \exp (3sq^{-1/2}) \ge \exp (3 q^{-1/2} (q \log q -1)/2 ) \ge 2^{10}, \end{aligned}$$

so $n \ge 11$. We use

28 $$\begin{aligned} \begin{aligned} J_{>n}(q^{-1/2})&\le 2^{-(n+1)} J(2q^{-1/2}), \\ J_{>n}(q^{-1})&\le (2 q^{1/2})^{-(n+1)} J(2q^{-1/2}), \\ J_{\le n}(1)&\le 1 + (q^{1/2}/2)^n ( J(2q^{-1/2}) -1), \\ (q^{1/2}+1)^2&\le (q^{1/2}/2)^n -1; \end{aligned} \end{aligned}$$

the first three are proved termwise, and the last follows from the inequality $(2u+1)^2 \le u^{11}-1$ for $u \,{:}{=}\,q^{1/2}/2 \ge 7^{1/2}/2$. By (28), the left side of (24) is at most

$$\begin{aligned}&2^{-(n+1)} J(2q^{-1/2}) + \frac{q^{n/2}}{2} (2q^{1/2})^{-(n+1)} J(2q^{-1/2}) \\&\qquad + \frac{q^{-n/2}}{2} \Bigl ( (q^{1/2}/2)^n J(2q^{-1/2}) + 1 - (q^{1/2}/2)^n \Bigr ) + \frac{q^{-n/2}}{2} \Bigl ( (q^{1/2}/2)^n - 1 \Bigr ) \\&\quad = 2^{-(n+1)}(2 + q^{-1/2}/2) \, J(2q^{-1/2}) \\&\quad \le 2^{1-n} J(2 q^{-1/2}) \\&\quad < \frac{1}{J(q^{-1/2})}, \end{aligned}$$

by hypothesis, so Proposition 9.3 applies.

Finally, suppose that $q \ge 16$ and $n > 3 q^{1/2} \log q - 1/2$. Then

29 $$\begin{aligned} s&\le (q \log q + 1)/2, \nonumber \\ \log \bigl (J(q^{-1/2}) J(2q^{-1/2}) \bigr )&\le 3 \Bigl ( \frac{q \log q +1}{2} \Bigr ) q^{-1/2} + \frac{q+1}{2} \Bigl (\frac{q^{-1}}{1-q^{-1/2}} + \frac{4 q^{-1}}{1-2q^{-1/2}} \Bigr ) \nonumber \\&\le (3 q^{1/2} \log q - 3/2) \log 2 \\&< (n-1) \log 2,\nonumber \end{aligned}$$

so (26) holds; to prove (29), check numerically for $16 \le q \le 100$ and for $q>100$ use

$$\begin{aligned}&\frac{3}{2} q^{-1/2} + \frac{q+1}{2} \Bigl (\frac{q^{-1}}{1-q^{-1/2}} + \frac{4 q^{-1}}{1-2q^{-1/2}} \Bigr ) + \frac{3}{2} \log 2 \\&\quad \le \frac{3}{2}(0.1) + q \Bigl (\frac{q^{-1}}{0.9} + \frac{4 q^{-1}}{0.8} \Bigr ) + \frac{3}{2} \log 2< 8 < (3 \log 2 - 3/2) q^{1/2} \log q. \end{aligned}$$

$$\square $$

Corollary 9.4 proves Theorem 1.13(b) for $q \ge 16$. Also, for each $q<16$ it provides an *n* such that all integers $\ge q^{n-1/2}$ are realizable, but too many integers remain to be checked one at a time. Therefore we describe a construction allowing us to realize larger intervals of integers all at once. The plan is to start with *h* such that ${\widehat{h}} = f_A$ for some *A* with $\#A({\mathbb {F}}_q)=m$, and then to replace *h* by $h + \sum _{i=r}^n c_i z^i$ for some *r* and small $c_i$ (and then adjust to make $p \not \mid {\widehat{h}}^{[n]}$ again); as the $c_i$ vary, we realize all integers in an interval.

### Construction 9.5

Suppose that we are given *q*, *n*, *m*, and a polynomial $h\in 1 + x {\mathbb {Z}}[x]$ of degree $<2n$ with ${\widehat{h}}(1)=m$ (given by Construction 9.1 or otherwise).

1. Compute the complex zeros of *h* and check that none of them are in *D*. (More precisely: Compute small balls containing the zeros, and check that none of them intersect *D*.)
2. Compute the complex zeros $\alpha$ of the derivative of *h*(*z*)*h*(1/(*qz*)), evaluate |*h*| at each $\alpha$ on the boundary $\partial D$, and let $\mu$ be the minimum of these values; see the proof of Lemma 9.6. (More precisely: Compute small balls around these zeros, and let $\mu$ be a lower bound for |*h*| on all these balls that intersect $\partial D$; if $h=1$, then let $\mu =1$.)
3. Let $\mu _{{{\,\textrm{ord}\,}}} = \mu - q^{-(n-1)/2} - ((q+1)/2) q^{-n/2}$; abort if $\mu _{{{\,\textrm{ord}\,}}} \le 0$.
4. Let *r* be the smallest positive integer $\le n+1$ such that $\sum _{i=r}^n \lfloor q/2 \rfloor q^{-i/2} < \mu _{{{\,\textrm{ord}\,}}}$.
5. Let $$N = \lfloor q/2 \rfloor \sum _{j=r}^n (q^{n-j}+1) = \lfloor q/2 \rfloor \left( \frac{q^{n-r+1}-1}{q-1} + (n-r+1) \right) $$.
6. Return the interval $\bigl [\,{\widehat{h}}(1) - N,{\widehat{h}}(1) + N\bigr ]$.

### Lemma 9.6

In Construction 9.5, if Steps 1 and 3 succeed, then every integer in the interval of Step 5 is $\#A({\mathbb {F}}_q)$ for some ordinary abelian variety of dimension *n* over ${\mathbb {F}}_q$.

### Proof

Suppose that *h* has no zeros in *D*. Then the minimum of |*h*| occurs on $\partial D$, where $|h|^2 = h(z) \, h(1/(qz))$, and this minimum occurs at a point where the derivative of $h(z) \, h(1/(qz))$ is 0. Thus $|h| \ge \mu$ on *D*.

Suppose that $H = h + \sum _{i=r}^n c_i z^i$ where $|c_i| \le q/2$ for all *i*, and $c_i \in {\mathbb {Z}}$ for all *i* except *n*, and $c_n \in \frac{1}{2}{\mathbb {Z}}$. The choice of *r* guarantees that $|H| > 0$ on *D*, even if we add $z^{n-1} - ((q+1)/2) z^n$ to *H* if necessary to make $p \not \mid {\widehat{H}}^{[n]}$, so ${\widehat{H}}(1)$ is realizable. To realize an integer ${\widehat{h}}(1) + M$ with $|M| \le N$, write *M* as $\sum _{j=r}^n c_j(q^{n-j} + 1)$ with $|c_j| \le \lfloor q/2 \rfloor$, $c_j \in {\mathbb {Z}}$ for all $j \ne n$, and $c_n \in \tfrac{1}{2} {\mathbb {Z}}$, by greedily choosing $c_r$, $c_{r+1}$, ..., one at a time. $\square$

### Proof of Theorem 1.13 and Remarks 1.16, 1.17, and 1.18

In this proof, given *q*, a positive integer is called realizable if it equals $\#A({\mathbb {F}}_q)$ for some ordinary abelian variety *A* over ${\mathbb {F}}_q$ with $f_A$ squarefree. The case $q=2$ is done by [7]. Criterion (27) of Corollary 9.4 proves Theorem 1.13(b) for $q \ge 16$. For each $q<16$, we numerically find $n \ge 2$ such that (25) holds; then we check smaller values of *n* to find the smallest $n_0$ such that (24) holds for all $n \ge n_0$. (It turns out that $n_0 \le 25$ for each $q<16$.) For $q \in \{11,13\}$, it turns out that $q^{3 \sqrt{q} \log q} > q^{n_0-1/2}$, which proves Theorem 1.13(b) for these *q*.

For $3 \le q \le 9$, we use variants of Construction 9.1 and 9.5 to realize all integers in an interval $[M_q,q^{n_0-1/2}]$. For $q \in \{8,9\}$, we have $M_q \le q^{3 \sqrt{q} \log q}$, which proves Theorem 1.13(b) for these *q*. For $q \in \{3,4,5,7\}$, we use the algorithm of [10] (implemented at https://github.com/kedlaya/root-unitary) to exhaust over the polynomials $f_A$ for abelian varieties *A* of dimension $\le 4$ to realize all integers $<M_q$ with the exception of those listed in Remarks 1.16, 1.17, and 1.18. Neither are these exceptions realized by abelian varieties of dimension $\ge 5$, because they are all less than $(\sqrt{q}-1)^{10}$. The calculations in this paragraph took 7.19 CPU hours on an Intel Core i7-9750 H CPU @ 2.60GHz. See https://github.com/edgarcosta/abvar-fq-orders for the code and further details. $\square$

Some calculations were checked against the database of isogeny classes of abelian varieties over finite fields in the L-functions and Modular Forms Database [3, 17].

## Data Availability

All computed data can be found at https://github.com/edgarcosta/abvar-fq-orders.

## Appendix A. Optimality of a potential function

### A.1. Polynomials

The goal of this appendix is to prove the following.

#### Proposition A.1

Choose *c* in the interval (0, 1). For $d \ge 1$, let ${\mathscr {F}}(d, c)$ be the set of complex polynomials *f* of degree *d* satisfying $f(0)=1$ and $|f(w)|^{1/d} \ge c$ for all $w \in {\mathbb {C}}_{\le 1}$. On $(-\infty ,1]$ define the decreasing continuous function

$$\begin{aligned} M(r) \,{:}{=}\,\frac{1 - r + \sqrt{(1 - r)^2 + 4rc^2}}{2}. \end{aligned}$$

- For any $f \in {\mathscr {F}}(d, c)$, we have
  30 $$\begin{aligned} |f(r)|^{1/d}&\ge M(r) \quad \, for all r \in [0,1], \end{aligned}$$
  31 $$\begin{aligned} |f(r)|^{1/d}&\le M(-r) \quad for all r \in [0,\infty ). \end{aligned}$$
- There exist polynomials $f_1,f_2,\ldots$ with $f_d \in {\mathscr {F}}(d,c)$ such that for every $r \in (-\infty ,1]$,
  32 $$\begin{aligned} \lim _{d \rightarrow \infty } |f_d(r)|^{1/d} = M(r). \end{aligned}$$
  (Thus (30) is asymptotically sharp, and (31) is too since $f_d(-z) \in {\mathscr {F}}(d,c)$.)

#### Remark A.2

For $r>1$, (30) is not always true: If $r>1$ and $c \le (1-r^{-d})^{1/d}$, the best lower bound in (30) is simply 0 since the function $f(z) \,{:}{=}\,1-(z/r)^d$ is in ${\mathscr {F}}(d,c)$.

#### Remark A.3

If *f* is in ${\mathscr {F}}(d,c)$, then so is *f*(*uz*) for any $u \in {\mathbb {C}}$ with $|u|=1$. Thus Proposition A.1 implies that (30) and (31) still hold if *f*(*r*) on the left is replaced by $f(r')$ for any complex number $r'$ satisfying $|r'|=r$.

Outside the trivial case $r = 0$ and the case $r = 1$, which was handled in detail in [13], Proposition A.1 appears to be new.

#### Remark A.4

Choose a prime power *q*, and take $c = q^{-1/4}$ and $r = q^{-1/2}$. Let $I_1, I_2, \dots$ be an increasing sequence of closed intervals with union $I_{attained }$. For each positive integer *k*, let $P_k$ be a polynomial constructed as in Proposition 6.2 from the interval $I_k$. Then the polynomials $P_k(q^{1/2}z) \in {\mathscr {F}}(\text {deg} P_k, c)$ have limits as in (32). In the other direction, Proposition A.1(a) shows that we could not hope to construct polynomials satisfying the conditions of Proposition 6.2 for intervals larger than $I_{attained }$.

### A.2. Potential functions

Given a nonconstant polynomial *f*, let $\mu$ be the uniform probability measure on the set of zeros of *f*, counted with multiplicity. Then $\log |f(z)|^{1/d}$ equals $\int \log |w-z|\,d\mu (w)$ minus a constant, so Proposition A.1 can be reformulated in terms of $\mu$. This suggests a generalization in which $\mu$ is allowed to be any compactly supported probability measure on ${\mathbb {C}}_{\ge 1}$. In fact, this generalization, formalized as Proposition A.6 below, is *equivalent* to Proposition A.1.

#### Definition A.5

([14, I.1]) Let $\Sigma$ be a compact subset of ${\mathbb {C}}$. Let ${\mathcal {M}}(\Sigma )$ be the set of (Borel) probability measures on ${\mathbb {C}}$ with support contained in $\Sigma$. For $\mu \in {\mathcal {M}}(\Sigma )$, define the potential function $U^{\mu } :{\mathbb {C}}\rightarrow {\mathbb {R}}\cup \{\infty \}$ by

$$\begin{aligned} U^{\mu }(z) \,{:}{=}\,\int _{{\mathbb {C}}} -\log |w - z|\, d\mu (w). \end{aligned}$$

For a polynomial *F* with nonzero constant term, the polynomial $f(z) \,{:}{=}\,F(z)/F(0)$ satisfies $f(0)=1$, as required in the definition of ${\mathscr {F}}(d,c)$. Analogously, we will consider $U^{\mu }(z)-U^\mu (0)$.

#### Proposition A.6

Choose *c* in (0, 1), and let ${\mathscr {M}}(c)$ be the set of probability measures $\mu$ with compact support contained in ${\mathbb {C}}_{\ge 1}$ such that

33 $$\begin{aligned} U^{\mu }(z) - U^\mu (0) \le - \log c \qquad \text {for all }z \in {\mathbb {C}}_{\le 1}. \end{aligned}$$

- For any $$\mu \in {\mathscr {M}}(c)$$,
  34 $$\begin{aligned} U^{\mu }(r) - U^{\mu }(0)&\le -\log M(r) \quad \text { for all } r \in [0,1], \end{aligned}$$
  35 $$\begin{aligned} U^{\mu }(r) - U^{\mu }(0)&\ge -\log M(-r) \quad \text { for all } r \in [0,\infty ). \end{aligned}$$
- Let $\Sigma _c$ be the arc $\{z \in {\mathbb {C}}: |z|=1 and |z-1| \le 2c\}$. There exists a measure $\mu _c \in {\mathscr {M}}(c)$ supported on $\Sigma _c$ such that for every $r \in (-\infty ,1]$,
  $$\begin{aligned} U^{\mu _c}(r) - U^{\mu _c}(0) = -\log M(r). \end{aligned}$$

#### Proof that Proposition A.6 implies Proposition A.1

If Proposition A.6(a) holds, apply it to the uniform probability measure $\mu$ on the zeros of $f \in {\mathscr {F}}(d,c)$ counted with multiplicity, and apply $x \mapsto e^{-x}$ to (34) and (35) to get Proposition A.1(a).

Now suppose in addition that Proposition A.6(b) holds. Fix $r \in [0,1]$. For $\lambda \in (0,1)$, Proposition A.6(a) (and rotational symmetry) shows that for $z \in {\mathbb {C}}_{\le \lambda }$,

36 $$\begin{aligned} U^{\mu _c}(z)-U^{\mu _c}(0) \le -\log M(\lambda ), \end{aligned}$$

which is *strictly* less than $-\log c$. For $s \in {\mathbb {C}}$, let $\delta _s$ be the Dirac probability measure supported at *s*. Since $\Sigma _c$ is compact, we can find subsets $S_d \subset \Sigma _c$ such that $\#S_d=d$ and $\frac{1}{d} \sum _{s \in S_d} \delta _s$ converges weakly to $\mu _c$ as $d\rightarrow \infty$. Let $p_d = \prod _{s \in S_d} (1-z/s)$, so

- $p_d$ has degree *d*, has all roots in ${\mathbb {C}}_{\ge 1}$, and satisfies $p_d(0)=1$; and
- on each compact subset of ${\mathbb {C}}{\setminus } \Sigma _c$, the sequence $-\log |p_d(z)|^{1/d}$ converges uniformly to $U^{\mu _c}(z)-U^{\mu _c}(0)$.

By (36) and uniform convergence, for any $\lambda <1$, if *d* is sufficiently large, then $-\log |p_d(z)|^{1/d} \le -\log c$ on ${\mathbb {C}}_{\le \lambda }$, so the polynomial $f_d(z) \,{:}{=}\,p_d(\lambda z)$ lies in ${\mathscr {F}}(d,c)$. Then $|f_d(r)|^{1/d} \rightarrow \exp (-(U^{\mu _c}(\lambda r)-U^{\mu _c}(0))) = M(\lambda r)$ uniformly on each compact subset of $(-\infty ,1]$. By repeating the argument for each $\lambda \in (0,1)$ to obtain $f_{d,\lambda }$, and then letting $\lambda$ tend to 1 sufficiently slowly with *d*, we obtain polynomials satisfying Proposition A.1(b). $\square$

The proof of Proposition A.6 occupies the rest of the appendix: (b) is proved in Section A.4, and the two inequalities of (a) are proved in Section A.5.

If $\mu$ is supported on the unit circle, then $U^\mu (0)=0$ by definition. In proving Proposition A.6(a), the following lets us assume that $\mu$ is supported on the unit circle.

#### Lemma A.7

Given a compactly supported probability measure $\mu$ on ${\mathbb {C}}_{\ge 1}$, there is a probability measure ${\widehat{\mu }}$ supported on the unit circle $\Sigma$ such that

$$\begin{aligned}U^{\mu }(z) - U^{\mu }(0) = U^{{\widehat{\mu }}}(z)\quad \text {whenever}\quad |z| < 1\end{aligned}$$

and

$$\begin{aligned}U^{\mu }(z) - U^{\mu }(0) \ge U^{{\widehat{\mu }}}(z)\quad \text {whenever}\quad |z| \ge 1.\end{aligned}$$

#### Proof

Write $\mu$ as a sum of nonnegative measures $\mu _\Sigma + \mu '$, where $\mu _\Sigma$ is supported on the circle and $\mu '(\Sigma )=0$. Apply “balayage” ([14, Theorem II.4.7]) to $\mu '$ to produce $\widehat{\mu '}$ supported on the circle, and let ${\widehat{\mu }} = \mu _\Sigma + \widehat{\mu '}$. $\square$

### A.3. Equilibrium measures

#### Definition A.8

Suppose that $\Sigma$ is of positive capacity, as defined in [14, (I.1.5)]; this holds if $\Sigma$ contains a line segment or circular arc of positive length, for example. The energy of $\mu \in {\mathcal {M}}(\Sigma )$ is $\int _{\Sigma } U^{\mu }(z) \, d\mu (z)$. There is a unique energy-minimizing measure $\mu \in {\mathcal {M}}(\Sigma )$, called the equilibrium measure on $\Sigma$. More generally, for any continuous function $Q :\Sigma \rightarrow {\mathbb {R}}$, there is a unique measure $\mu$ in ${\mathcal {M}}(\Sigma )$ minimizing the weighted energy

$$\begin{aligned} E_Q(\mu ) \,{:}{=}\,\int _{\Sigma } \big (U^{\mu }(z) + 2 \, Q(z)\big )\, d\mu (z), \end{aligned}$$

and this $\mu$ is called the weighted equilibrium measure for *Q* on $\Sigma$.

From now on, $\Sigma$ denotes the unit circle, and $\kappa :{\mathbb {C}}^\times \rightarrow {\mathbb {C}}$ denotes the rational function $\kappa (z) \,{:}{=}\,z+z^{-1}$, which maps $\Sigma$ onto the interval $[-2,2]$.

#### Lemma A.9

- The map $\mu \mapsto \kappa _* \mu$ sending measures to their pushforwards under $\kappa$ is a bijection from the set of complex-conjugation-invariant probability measures on the unit circle $\Sigma$ to ${\mathcal {M}}([-2,2])$.
- For $\mu$ as in (a), we have $U^{\kappa _*\mu }(\kappa (z)) = 2 \, U^\mu (z) + \log |z|$ for all $z \in {\mathbb {C}}^\times$.
- Let $\Sigma '$ be a positive-capacity complex-conjugation-invariant compact subset of $\Sigma$. Let $\alpha \in {\mathbb {R}}$ and $r \in {\mathbb {R}}^\times {\setminus } \Sigma '$. Under $\kappa _*$, the equilibrium measure on $\Sigma '$ for weight $Q(z) \,{:}{=}\,\alpha \log |z - r|$ corresponds to the equilibrium measure on $\kappa (\Sigma ')$ for weight $R(z) \,{:}{=}\,\alpha \log |z - \kappa (r)|$.

#### Proof

- The map $\kappa$ induces an isomorphism from the $\sigma$-algebra of complex-conjugation-invariant Borel subsets of $\Sigma$ to the $\sigma$-algebra of Borel subsets of $[-2,2]$.
- For $w,z \in {\mathbb {C}}^\times$ we have
  37 $$\begin{aligned} \kappa (w)-\kappa (z) = -\frac{1}{z} (w-z)(w^{-1}-z). \end{aligned}$$
  The claim follows by applying $-\log |\;|$ and integrating against $d\mu (w)$; the integrals of $\log |w-z|$ and $\log |w^{-1}-z|$ are equal since $\mu$ is invariant under $w \mapsto {\bar{w}}=w^{-1}$.
- Renaming variables in (37) yields $R(\kappa (z)) = 2 \, Q(z) - \alpha \log |r|$. By symmetry, the only measures on $\Sigma '$ we need to consider are those that are complex-conjugation-invariant. For such $\mu$,
  $$\begin{aligned} E_R(\kappa _* \mu )&= \int _{\Sigma '} \big ( U^{\kappa _* \mu }(\kappa (z)) + 2 \, R(\kappa (z)) \big ) \, d\mu (z) \\&= \int _{\Sigma '} \big ( 2 \, U^{\mu }(z) + 4 \, Q(z) - 2 \alpha \log |r| \big ) \, d\mu (z)\\  &\qquad \quad (by (b), since |z|=1 on \Sigma ' \subseteq \Sigma ) \\&= 2 \, E_Q(\mu ) - 2 \alpha \log |r|, \end{aligned}$$
  so the $\mu$ that minimizes $E_R(\kappa _*\mu )$ is the same as the $\mu$ that minimizes $E_Q(\mu )$. $\square$

### A.4. The extreme measure

Let *c* and $\Sigma _c$ be as in Proposition A.6. Let $\mu _c$ be the equilibrium measure on $\Sigma _c$. Lemma A.10 below shows that $\mu _c$ satisfies the requirements of Proposition A.6(b).

#### Lemma A.10

- We have $$U^{\mu _c}(0)=0$$.
- The function $U^{\mu _c}(z)$ is $${\left\{ \begin{array}{ll} -\log c &  for z \in \Sigma _c, \\ \le -\log c &  for z \not \in \Sigma _c. \end{array}\right. }$$
- For all $r \in (-\infty ,1]$, we have $U^{\mu _c}(r) = -\log M(r)$.

#### Proof

- This holds for any measure supported on the unit circle, by definition of the potential.
- By [12, Table 5.1], the capacity of the equilibrium measure on the circular arc $\Sigma _c$ is *c*, so its energy is $-\log c$. The inequality outside $\Sigma _c$ follows from [14, pp. 53-54]. The equality on $\Sigma _c$ follows from the fact that the points on $\Sigma _c$ are regular points for the Dirichlet problem on ${\mathbb {C}}{\setminus } \Sigma _c$, as can be checked from [14, Theorem I.4.6].
- By similar right triangles, the real part of either endpoint of $\Sigma _c$ is at distance $2c^2$ from 1, so $\kappa (\Sigma _c) = 2[1-2c^2,1] = [2-4c^2,2]$. Let $\ell (z) \,{:}{=}\,c^2 z + 2-2c^2$, so $\ell ([-2,2]) = [2-4c^2,2]$. Let $\mu _\Sigma$ be the uniform probability measure on $\Sigma$, so $U^{\mu _{\Sigma }}(z)=0$ on ${\mathbb {C}}_{\le 1}$ [14, Example 0.5.7]. By Lemma A.9(c), $\kappa _*\mu _c$ and $\kappa _*\mu _\Sigma$ are the equilibrium measures on $[2-4c^2,2]$ and $[-2,2]$, respectively, so $\kappa _*\mu _c = \ell _* \kappa _* \mu _\Sigma$. Given $r \in (0,1]$, define $r' \in (0,1]$ by $\kappa (r) = \ell (\kappa (r'))$. Applying $U^{\kappa _*\mu _c} = U^{\ell _* \kappa _* \mu _\Sigma }$ yields
  $$\begin{aligned} U^{\kappa _*\mu _c}(\kappa (r))&= U^{\kappa _* \mu _\Sigma }(\kappa (r')) - \log c^2  &   (since \,\, \ell \text { scales distances by}\,\, c^2) \\ 2 \, U^{\mu _c}(r) + \log r&= (2 \, U^{\mu _{\Sigma }}(r') + \log r') - \log c^2  &   (by Lemma ~A.9\text {(b) twice)} \\ U^{\mu _c}(r)&= \tfrac{1}{2} \log (r'/r) - \log c  &   (since \,\, U^{\mu _{\Sigma }}(z)=0 \,\,\text {on}\,\, {\mathbb {C}}_{\le 1}) \\&= - \log M(r)  &   \!\!\!\!\!\!\!\!\!\!\!\!\!\!\!\!\!\!\!\!\!\!\!\!\!\!\!\!\!\!(algebraic computation yields r' \, M(r)^2 = r c^2). \end{aligned}$$
  To extend to $(-\infty ,1]$, observe that $U^{\mu _c}(r)$ and $-\log M(r)$ are real analytic on $(-\infty ,1)$. $\square$

### A.5. Proof of optimality

The idea for proving inequality (34) is that it should be a nonnegative linear combination (really an integral) of the inequalities (33). The “coefficients” of the linear combination are given by a measure $\nu _\alpha$ belonging to a family that we describe now. The $r=0$ and $r=1$ cases of (34) follow from $M(0)=1$ and $M(1)=c$, so we assume $r \in (0,1)$. For $\alpha \in {\mathbb {R}}_{\ge 0}$, let $\nu _\alpha$ be the equilibrium measure on $\Sigma$ for weight $Q_{\alpha }(z) \,{:}{=}\,\alpha \log |z - r|$.

#### Lemma A.11

- For every $\alpha \ge 0$, there exists $\theta (\alpha ) \in (0, \pi ]$ such that ${{\,\textrm{supp}\,}}(\nu _{\alpha })$ is the arc $\{e^{it} :|t| \le \theta (\alpha )\}$.
- The function $\theta$ is decreasing and continuous. Also, $\theta (0) = \pi$ and $\lim _{\alpha \rightarrow \infty } \theta (\alpha ) = 0$.
- Let $\alpha$ be such that ${{\,\textrm{supp}\,}}(\nu _\alpha ) = \Sigma _c$. Then there is a constant *C* such that
  38 $$\begin{aligned} U^{\nu _\alpha }(z) + Q_\alpha (z) \; is \; {\left\{ \begin{array}{ll} C &  for all z \in \Sigma _c, \\ \ge C &  for all z \in \Sigma {\setminus } \Sigma _c. \end{array}\right. } \end{aligned}$$

#### Proof

The literature contains similar results for an interval; to use them, we push forward by $\kappa$. By Lemma A.9(c), $\kappa _*\nu _\alpha$ is the equilibrium measure on $[-2,2]$ for weight $Q_1(z) \,{:}{=}\,\alpha \log |z-\kappa (r)|$.

- We need to prove that ${{\,\textrm{supp}\,}}(\kappa _* \nu _\alpha ) = [2\cos (\theta (\alpha )),2]$ for some $\theta (\alpha ) \in (0,\pi ]$. Pushing forward $\kappa _* \nu _\alpha$ by $z \mapsto 2-z$ gives the equilibrium measure on [0, 4] for weight $Q_2(z) \,{:}{=}\,\alpha \log |\kappa (r) - 2 + z|$. The function
  $$\begin{aligned}x \, Q_2'(x) = \frac{\alpha x}{\kappa (r) - 2 + x}\end{aligned}$$
  is increasing on [0, 4], so [14, Theorem IV.1.10(c)] implies that the support is an interval. The corresponding interval for $\kappa _* \nu _\alpha$ is contained in $[-2,2]$, and must contain 2 since otherwise we could translate the measure right to reduce the weighted energy.
- By [14, Theorem IV.1.6(f)], ${{\,\textrm{supp}\,}}(\nu _\alpha )$ is decreasing and continuous,^1^ so $\theta$ is too. Since $\nu _0$ is the uniform measure on $\Sigma$, we have $\theta (0)=\pi$. For fixed $\epsilon>\epsilon '>0$, if $\kappa _*\nu _\alpha$ has any mass to the left of $2-\epsilon$, redistributing it according to the equilibrium measure on $[2-\epsilon ',2]$ increases the energy by *O*(1) but decreases the contribution from the weight by at least a positive constant times $\alpha$, so if $\alpha$ is sufficiently large, $\kappa _*\nu _\alpha$ cannot have such mass; in other words, ${{\,\textrm{supp}\,}}(\kappa _*\nu _\alpha ) \subset [2-\epsilon ,2]$ for sufficiently large $\alpha$. This holds for every $\epsilon$, so $\lim _{\alpha \rightarrow \infty } \theta (\alpha ) = 0$.
- The number $\alpha$ exists by (b). Let *C* be the modified Robin constant for $Q_\alpha$ [14, p. 27]. By [14, Theorem I.1.3(d,f)], (38) holds outside a zero-capacity subset of $\Sigma$. On the other hand, the points in $\Sigma$ are regular points for the Dirichlet problem in $\overline{{\mathbb {C}}} {\setminus } \Sigma$ by Wiener’s theorem [14, Theorem I.4.6], so [14, Theorem I.5.1(iv$'$)] implies that $U^{\nu _\alpha }$ is continuous on $\Sigma$, as is $Q_\alpha$. Thus (38) holds on all of $\Sigma$. $\square$

#### Proof of (34)

By Lemma A.7, we may assume that $\mu$ is supported on the unit circle $\Sigma$, so $U^\mu (0)=0$. Given $r \in (0,1)$, let $\alpha$ be as in Lemma A.11(c). Then

$$\begin{aligned} -U^{\mu }(r)&= \frac{1}{\alpha } \int _{\Sigma }Q_{\alpha }(z) \, d\mu (z)  &   (since \,\, Q_{\alpha }(z) = \alpha \log |z-r|) \\&\ge \frac{1}{\alpha } \left( C - \int _{\Sigma } U^{\nu _\alpha }(z) \, d\mu (z) \right)  &   (by the inequality in ~(38)) \\&= \frac{C}{\alpha } + \frac{1}{\alpha }\int _{\Sigma _c} \int _{\Sigma } \log |z - w|\, d\mu (z) \, d\nu _\alpha (w)  &   (by definition of U^{\nu _\alpha }) \\&= \frac{C}{\alpha } - \frac{1}{\alpha }\int _{\Sigma _c} U^{\mu }(w) \, d\nu _\alpha (w)  &   (by definition of \,\, U^{\mu }) \\&\ge \frac{C}{\alpha } + \frac{1}{\alpha } \log c  &   (by (33)\,\, \text {with}\,\, U^\mu (0)=0). \end{aligned}$$

When $\mu$ is $\mu _c$, Lemmas A.11(c) and A.10(b) show that both inequalities in this sequence are sharp, so

$$\begin{aligned} -U^{\mu _c}(r) = \frac{C}{\alpha } + \frac{1}{\alpha } \log c \le -U^{\mu }(r). \end{aligned}$$

Thus $U^{\mu }(r) - U^{\mu }(0) = U^{\mu }(r) \le U^{\mu _c}(r) = - \log M(r)$, by Lemma A.10(c). $\square$

#### Proof of (35)

For $\beta \in {\mathbb {R}}_{\ge 0}$, let $\nu '_\beta$ be the equilibrium measure on $\Sigma$ for weight $R_{\beta }(z) \,{:}{=}\,-\beta \log |z + r|$. As in Lemma A.11, there exists $\beta >0$ and a real constant *D* such that ${{\,\textrm{supp}\,}}(\nu '_{\beta }) = \Sigma _c$ and

$$\begin{aligned} U^{\nu '_{\beta }}(z) + R_{\beta }(z)\; is \; {\left\{ \begin{array}{ll} D &  for all z \in \Sigma _c, \\ \ge D &  for all z \in \Sigma {\setminus } \Sigma _c. \end{array}\right. } \end{aligned}$$

We may replace $\mu$ by the ${\widehat{\mu }}$ given by Lemma A.7, which implies that $U^\mu (r) - U^\mu (0) \ge U^{{\widehat{\mu }}}(r)$ for every $r \in [0,\infty )$. The rest of the proof is entirely analogous to the proof of (34). $\square$
